# Hybrid Approach to Estimation of Underreporting of Tuberculosis Case Notification in High-Burden Settings With Weak Surveillance Infrastructure: Design and Implementation of an Inventory Study

**DOI:** 10.2196/22352

**Published:** 2021-03-15

**Authors:** Ellen M H Mitchell, Olusola Adedeji Adejumo, Hussein Abdur-Razzaq, Chidubem Ogbudebe, Nkem Chukwueme, Samson Bamidele Olorunju, Mustapha Gidado

**Affiliations:** 1 Tropical Infectious Diseases Department of Public Health Institute for Tropical Medicine Antwerp Belgium; 2 Mainland Hospital Yaba Lagos Nigeria; 3 Health Research Unit Lagos State Ministry of Health Lagos Nigeria; 4 KNCV TB Foundation Nigeria Abuja Nigeria; 5 New York Medical College New York, NY United States; 6 Department of Epidemiology and Medical Statistics Faculty of Public Health University of Ibadan Ibadan Nigeria; 7 KNCV TB Foundation Koninklijke Centrale Vereniging tot bestrijding der Tuberculose (KNCV) The Hague Netherlands

**Keywords:** tuberculosis, notification, integrated disease surveillance reporting, private sector, inventory study, public health surveillance, epidemiology, infectious disease reporting, infectious disease notification

## Abstract

**Background:**

The greatest risk of infectious disease undernotification occurs in settings with limited capacity to detect it reliably. World Health Organization guidance on the measurement of misreporting is paradoxical, requiring robust, independent systems to assess surveillance rigor. Methods are needed to estimate undernotification in settings with incomplete, flawed, or weak surveillance systems. This study attempted to design a tuberculosis (TB) inventory study that balanced rigor with feasibility for high-need settings.

**Objective:**

This study aims to design a hybrid TB inventory study for contexts without World Health Organization preconditions. We estimated the proportion of TB cases that were not reported to the Ministry of Health in 2015. The study sought to describe TB surveillance coverage and quality at different levels of TB care provision. Finally, we aimed to identify structural-, facility-, and provider-level barriers to notification and reasons for underreporting, nonreporting, and overreporting.

**Methods:**

Retrospective partial digitalization of paper-based surveillance and facility records preceded deterministic and probabilistic record linkage; a hybrid of health facilities and laboratory census with a stratified sampling of HFs with no capacity to notify leveraged a priori knowledge. Distinct extrapolation methods were applied to the sampled health facilities to estimate bacteriologically confirmed versus clinical TB. In-depth interviews and focus groups were used to identify causal factors responsible for undernotification and test the acceptability of remedies.

**Results:**

The hybrid approach proved viable and instructive. High-specificity verification of paper-based records in the field was efficient and had minimal errors. Limiting extrapolation to clinical cases improved precision. Probabilistic record linkage is computationally intensive, and the choice of software influences estimates. Record absence, decay, and overestimation of the private sector TB treatment behavior threaten validity, meriting mitigation. Data management demands were underestimated. Treatment success was modest in all sectors (*R*=37.9%–72.0%) and did not align with treatment success reported by the state (6665/8770, 75.99%). One-fifth of TB providers (36/178, 20%) were doubtful that the low volume of patients with TB treated in their facility merited mastery of the extensive TB notification forms and procedures.

**Conclusions:**

Subnational inventory studies can be rigorous, relevant, and efficient in countries that need them even in the absence of World Health Organization preconditions, if precautions are taken. The use of triangulation techniques, with minimal recourse to sampling and extrapolation, and the privileging of practical information needs of local decision makers yield reasonable misreporting estimates and viable policy recommendations.

## Introduction

### Background

The need to assess the quality and coverage of infectious disease surveillance has increased exponentially in recent years, as emerging infections and antimicrobial resistance crises have highlighted the perils of incomplete, paper-based surveillance systems and lax patient follow-up [1-3]. Tuberculosis (TB) inventory studies are a means to assess the level of misreporting of TB cases in a defined geographical area. Inventory studies help policy makers distinguish between the volume of people with TB who were never reached or treated and those who were treated and simply never reported. Getting these proportions right is critically important to understanding and addressing a country’s TB epidemic.

### Objectives

The primary aim of this study was to design a methodology that is adequate to determine the magnitude and scope of TB misreporting in Lagos state, a megacity with a highly dynamic private sector. To achieve this, it was necessary to adapt and test an unorthodox approach reflective of this context’s unique advantages and challenges. Local stakeholders had a pragmatic need to understand the root causes of undernotification and potential solutions’ palatability. Thus, the objectives of this study were to determine the following: (1) number (and proportion) of TB cases treated that were not reported to the Ministry of Health in 2015 among engaged and unengaged facilities; (2) number, proportion, and type of TB facilities that did not comply with the obligation to notify TB cases in 2015 (public and private); (3) characteristics of unnotified versus notified patients with TB; (4) surveillance coverage and quality at different levels of the TB surveillance system; (5) structural-, facility-, and provider-level barriers to notification and reasons for underreporting, nonreporting, and overreporting; and (6) actionable recommendations to improve notification and surveillance quality at the state, local government area (LGA), and facility levels.

As important as inventory studies can be used to shape a country's TB control strategy, the means of obtaining this information is far from simple. In 2012, the World Health Organization (WHO) articulated a series of 7 essential ingredients that must be in place before attempting an inventory study [4]:

1. Case-based data with reliable personal identifiers for record linkage
2. Use of standardized TB case definitions across all care providers
3. Ability to map all care providers outside the existing National Tuberculosis Program (NTP) network
4. Ability to convince all care providers to participate (ie, minimal refusal)
5. Expertise in sampling design, data management, and data analysis
6. At least three independent data sources and sampling of 50% of country areas
7. Statistical research capacity and funding for a research study.

At the time of publication of the WHO inventory study guide in 2012, virtually none of the countries with high TB incidence could fulfill all prerequisites, effectively precluding the measurement of undernotification in the settings where it was most urgent to undertake it. Moreover, developing some of the *essential items* would introduce an ascertainment bias because these elements are known to have a positive association with infectious disease reporting behavior (eg, case-based data systems and constructive relationships with non-NTP care providers) [5]. Universal private sector consent for participation and zero cross-border care seeking were ethically and practically untenable; thus, it was necessary to develop methods to assess the magnitude of biases because of refusal and in- and out-migration.

We chose to combine and adapt methodologies from several WHO-recommended study designs and innovate as needed to design an efficient solution appropriate for high-burden settings with weak surveillance systems. In this paper, we describe the Lagos hybrid method, report how assumptions and techniques are performed, and articulate lessons learned for future replication in high-burden settings where estimates of misreporting are necessary.

## Methods

### Study Setting

In 2015, Nigeria was considered by many to be at high risk for both under- and overnotification of TB cases because of its sprawling private health sector; yawning treatment coverage gap; ambitious donor-driven case detection targets; and fractured, overlapping infectious disease surveillance systems [6-10]. Nigeria continues to rank near the top of the list of TB high-burden countries in terms of unmet need for TB treatment, with 91,354 TB cases notified and a TB prevalence rate of 330 per 100,000 people in 2014 [11]. Results from Nigeria’s 2012 National TB Prevalence Survey indicated an approximate case detection rate (CDR) of 17%; this would mean that the 100,401 notified TB cases in 2013 represented only 17% of the total estimated 591,000 new TB cases occurring in the same year [7]. According to the 2015 Global TB Report, the WHO estimated that the CDR decreased to 15% [2]. According to projections based on the 2006 census, Lagos’s estimated population is 12.5 million, with an average density of 4990 individuals per square kilometer. More than 65% of Lagos’s population lives below the poverty line.

Applying the WHO estimate, the Lagos treatment coverage is 11% because less than 10,000 cases were reported out of the estimated 90,000 cases. Lagos is the largest megacity in Africa in terms of population, divided into 20 administrative districts (referred to as LGA). Border areas are not consistently demarcated, and administrative taxation, transport, and environmental arrangements reflect a functional interdependence between states Ogun and Lagos.

Although Nigeria presents challenges for evaluating disease surveillance systems, it also offers strategic advantages for the study of misreporting such as parallel, independent official infectious disease reporting systems, multiple sources of TB diagnostic and treatment data, and rigorous recent censuses of the sprawling private sector [12]. The study design was tailored to leverage available data sources and triangulates to improve precision. Specifically, we started from or expanded the WHO inventory study recommendations: (1) retrospective digitalization of paper-based records to create electronic case-based records; (2) use of high-specificity on-site verification procedures to reduce data entry and respondent burden; (3) estimation of the volume of private sector provision of clinical versus bacteriological TB diagnosis using direct and indirect methods; (4) use of buffer zone sensitivity analysis to estimate misreporting in an area *without* well-defined, closed geographical boundaries; (5) estimation of misreporting between administrative levels of the notification system; and (6) study of the underlying rationales and solutions to suboptimal notification behaviors in addition to its quantification.

**Figure 1:**
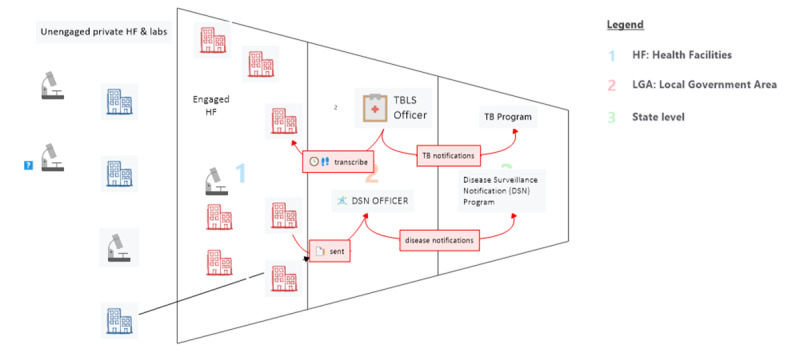
TB surveillance systems in Nigeria in 2015. TB: tuberculosis; TBLS: tuberculosis and leprosy supervisor.

Figure 1 describes the 2 parallel TB surveillance systems at 2 administrative levels. In parallel to the Lagos TB program, TB outpatient and inpatient treatments are also notifiable through a state-wide monthly disease reporting system known as the Integrated Disease Surveillance and Response (IDSR). The State Tuberculosis and Leprosy Control Program (STBLCP) is the authoritative department within the Lagos State Ministry of Health on TB surveillance matters.

### Study Design

**Figure 2:**
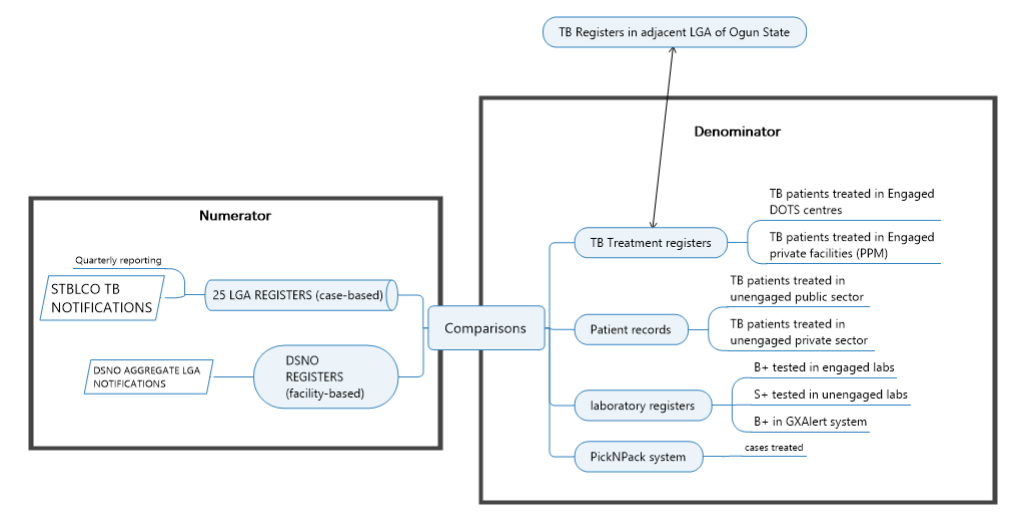
Map of primary data sources for the estimation of the magnitude of underreporting. B+: bacteriologically confirmed TB; DOTS: Directly Observed Treatment Short-Course; DSNO: disease surveillance and notification officer; LGA: local government area; PPM: public-private mix; S+ : smear positive; STBLCO: state tuberculosis and leprosy control officer; TB: tuberculosis.

The WHO recommends that inventory study designs in settings without case-based electronic surveillance employ prospective designs in a representative sample of diagnostic sites [4]; however, the addition of data collection systems with standardized case definitions and capacity building required to measure surveillance behavior can introduce bias because these tend to improve surveillance behavior and reporting [13]. Retrospective designs are only recommended in settings with multiple preexisting electronic case-based data sets with standardized case definitions across all providers, an improbable scenario [11]. Therefore, we attempted a hybrid design, quality-assured digitalization of paper-based notifications, and high-specificity verification of patient source documents to avoid full transcription of source documents in the field. The study triangulated TB case-based data derived from 6 sources instead of 3 (Figure 2).

1. STBLCP aggregate TB case notifications by LGA (State totals)
2. Case-based TB notifications at LGA level (LGA registers)
3. Smear and/or GeneXpert positive diagnostic test results from engaged laboratories in 2015
4. Smear and/or GeneXpert positive diagnostic test results from unengaged standalone laboratories.
5. Patients with TB with Lagos addresses diagnosed or treated in DOTS facilities in 3 contiguous LGA in Ogun in 2015 (abstracted from facility register by data collectors)
6. Patients with TB with Ogun addresses diagnosed or treated in DOTS facilities in Lagos.

### Sampling

The sampling frames were derived from 5 facility databases: Health Facility Monitoring and Accreditation Agency (HEFAMAA), Millennium Development Goals, Strengthening Health Outcomes through the Private Sector, the State TB program's list of DOTS providers, and the Pick'N'Pack drug management system records [12,14]. We used 2 lists of DOTS centers maintained by the State TB program to create the sampling frame of the engaged DOTS facilities; this included 217 public engaged facilities and 98 private providers involved in the public-private mix (PPM) scheme. For the purposes of the study, we considered a public facility *engaged* if it was listed on the official TB program DOTS facility list or if it was found in the list of facilities in the TB drug commodity management system (Pick'N'Pack) [15]. We aimed to include a census of all engaged facilities; 3 sites reported that they had been engaged in 2016, but these were still classified according to their 2015 engagement status.

Although the PPM program in Lagos is one of the most established and well studied in Nigeria, the actual size of the official engaged PPM program in Lagos is modest, involving 96 private HFs or less than 5% of Lagos's private HF [16-19]. Accurate estimation of the unengaged HFs sample frame was crucial to avoid over- or underestimation and underreporting in the extrapolation phase. Multimedia Appendix 1 [20-23] describes the samples and sampling in detail.

There were 131 public HFs that did not provide TB services and were considered *unengaged*. Among them were 27 sites that had historically offered TB care but had ceased providing TB diagnosis and/or treatment in 2015 because of staffing, managerial, or infrastructure constraints. Some had been classified by the TB program as *semidormant* or *dormant* and had been removed from official lists of DOTS centers. As the study was retrospective, we knew that interventions were planned during the 2015 or 2016 period to resume TB services at dormant sites. Therefore, we sampled 27 nominally *unengaged* public facilities that had historically been DOTS facilities, had laboratory capacity, or had received TB drugs in the recent past. A list maintained by the State TB program of engaged laboratories participating in TB activities in 2015 was used as the sampling frame. Engaged laboratories were trained and equipped to conduct smear microscopy and/or GeneXpert tests. All the engaged laboratories were eligible as they were likely to have a high volume of TB diagnoses, and sampling could have introduced bias.

The HEFAMAA database of 349 registered laboratories was deduplicated and compared with the standalone laboratories in the DOTS database to identify 272 unengaged private laboratories.

### Eligibility Criteria of HFs

Facilities were invited to participate if they were functioning as health service providers at the time of the survey (mid-2017) and if they met the eligibility criteria. The eligibility criteria for the inclusion of HFs in the study are listed in Textbox 1.

Laboratories were eligible for inclusion as engaged if they were standalone diagnostic centers that did not treat and were not physically housed inside or otherwise connected to an HF; the laboratories inside HFs were searched as part of verification exercises for engaged and unengaged HFs.

**Eligibility and exclusion criteria of the health facilities.**

1. Having at least one registered nurse or medical doctor employed
2. If public, health facilities had to have access to tuberculosis drugs to be considered as treating patients with tuberculosis.

The following health providers or facilities were excluded:

- Community pharmacies
- Patent medical vendors [24]
- Mobile clinics whose location cannot be determined a priori
- Dental and eye clinics
- Dialysis center
- Physiotherapy clinics
- Corporate health facilities

### Sampling Rationale

**Figure 3:**
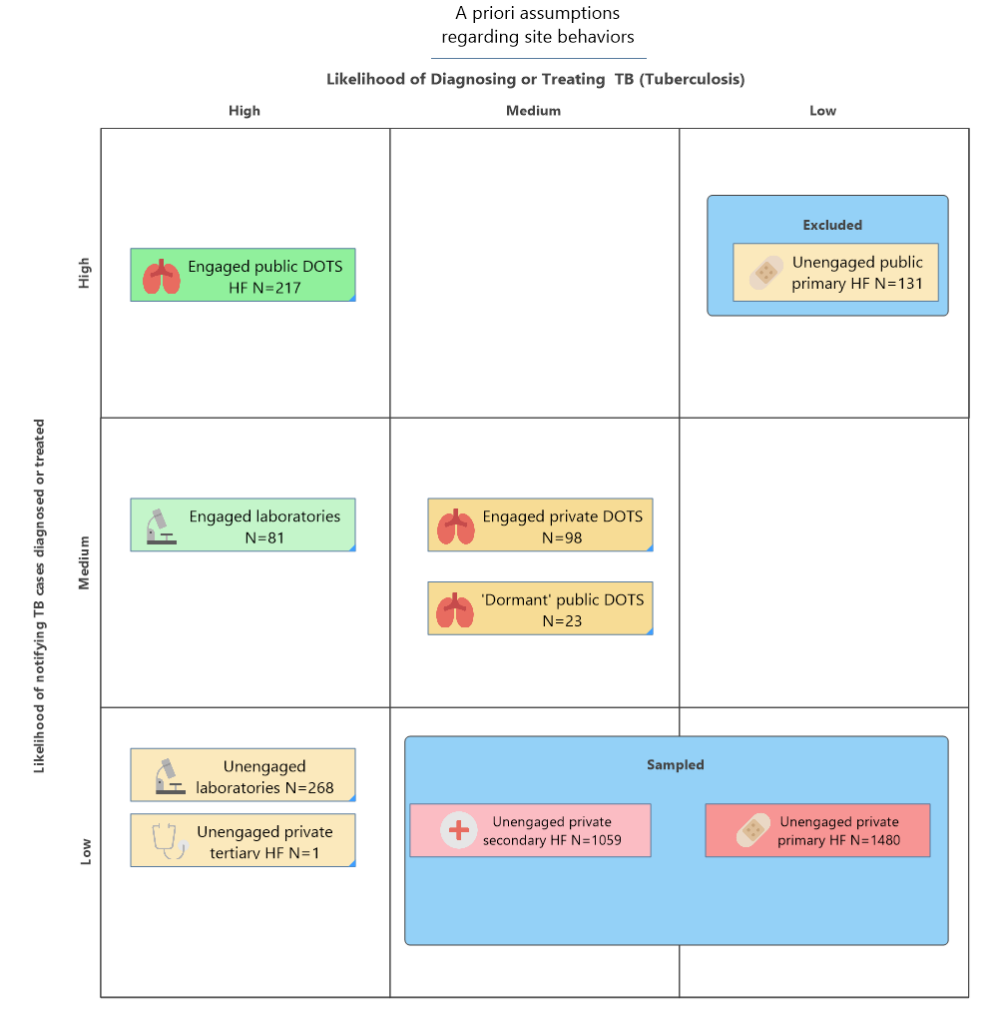
Behavioral assumptions driving the decision to census or sample health facility strata. DOTS: directly observed therapy short-course; HF: health facilities; TB: tuberculosis.

The choice to census or sample a facility stratum was based on 3 factors: the size of the stratum (a proxy for the affordability of a census), probability of offering TB treatment, and the probability of TB case notification capacity (Figure 3). Previous studies have demonstrated that the volume of TB cases treated in engaged DOTS centers and engaged labs were variable and dynamic over time, TB notification capacity was present, and misreporting was expected to be minimal, so a census of these strata was prudent to minimize error. All facilities engaged as of 2015 were eligible and invited to participate—public DOTS, private DOTS, and laboratories. There was not enough a priori information about TB diagnostic capacity or volume in unengaged private laboratories to sample them in an unbiased manner, so we chose to census all the unengaged laboratories. Finally, there were 131 unengaged public facilities without staffing, diagnostic capacity, or drugs to treat TB. We excluded these facilities from the study with the exception of 23 public facilities that had offered TB services in the past or were undergoing TB training and engagement during 2015; they were not on any official lists of engaged DOTS centers, and their status was ambiguous. Therefore, we purposefully sampled all sites that had historically been DOTS facilities, had laboratory capacity, or had been targeted for resuming TB diagnosis and/or treatment.

Sampling was necessary for the unengaged private HFs sector because it was not economically feasible to include 2634 HFs. A previous study showed that 32% to 36% of private providers offered TB treatment, and the probability of offering TB care was related to the facility level [12]. Therefore, stratified sampling by level with probability proportional to size appeared viable. We hypothesized that TB case underreporting behavior was common, if not universal, among unengaged private providers. Unengaged private providers had no access to the required NTP recording and reporting (R+R) forms, and the TB program did not accept TB notifications that did not employ national forms and guidelines. We decided to stratify the sample by the HF level, with oversampling of secondary level facilities for 3 reasons. First, we reasoned that larger facilities were more likely to have a more formalized R+R system in place for Disease Surveillance and Notification Officer and HEFAMAA certification, so we assumed slightly lower levels of undernotification behavior and a bigger sample needed to detect it. Second, more complex facilities have more departments with staff and equipment to diagnose TB but less experience reporting it. Finally, Nigerians’ recourse to hospitals for TB care is well documented and obliged a robust estimate in this sector. Among 1142 individuals interviewed for health-seeking behavior through Nigeria’s 2012 National TB Prevalence Survey, 45% first sought care from a hospital [20].

### Sample Size Calculation for Unengaged Private HFs

The sample size formula recommended by WHO frames TB cases as the sampling unit and requires previous knowledge of the harmonic mean of the cluster size (ie, the average number of TB cases found in HFs) and the coefficient of between-cluster variation—both parameters unlikely to be known to investigators researching the mostly unregulated private health sectors of high-burden countries. As we sampled only unengaged private HFs, HF was our sampling unit. Applying the standard sample size formula for estimating the population proportion (ie, the proportion of HFs notifying) with specified relative precision, 327 HFs were required (Table 1). Therefore, 380 replacement HFs were drawn from the frame to be substituted in the event of refusal, closures, and other eventualities. We drew the sample proportionally to the distribution of HFs among the LGAs. Oversampling secondary level facilities was prudent as this stratum had a higher likelihood of TB provision, so even small errors could significantly impact estimates of the overall magnitude of undernotified TB treatment. Although missing TB notifications was unlikely, undercounting treated TB cases in the unengaged private sector was the major validity threat to the study. We also had to power the study such that we would accrue enough unnotified TB cases to describe their characteristics and identify any predictors of non-notification. We aimed to recruit a minimum of 112 unengaged private HF patients with TB treatment documentation.

**Table 1:** Overview of health facility and laboratory sampling plan.

Type of facility			Numbers of facilities	Sampled quotient, n (%)	Type of sample
**Health facility**					
	Public engaged		217	217 (100.00)	Census (take all)
	Private engaged		98	98 (100.00)	Census (take all)
	Public unengaged		131	23 (17.55)	Purposive (take all dormant/former DOTS centres)
	**Private unengaged**		2634	327 (12.41)	Stratified sample with selection proportional to size sampled with replacement
		Primary	1480	141 (9.53)	N/A^a^
		Secondary	1059	186 (17.56)	N/A
	Total		3080	665 (21.59)	N/A
**Laboratory**					
	Public engaged		43	43 (100.00)	Census (take all)
	Private engaged		33	33 (100.00)	Census (take all)
	Public unengaged		4	4 (100.00)	Census (take all)
	Private unengaged		268	268 (100.00)	Census (take all)
	Total		349	349 (100.00)	N/A

^a^N/A: not applicable.

### TB Case Definition

For this study’s purposes, the definition of a TB case was broad and subdivided into 2 groups: (1) patients with bacteriologically confirmed (B+) TB who had one or more positive smear microscopy test results and/or a positive GeneXpert test result and (2) patients with clinical TB who were diagnosed by a clinician via a chest x-ray, children diagnosed with symptoms compatible with TB and responsive to treatment, and patients of any age treated by a formal medical provider for drug-sensitive TB for a minimum of 1 month, regardless of the site or type of TB or means of diagnosis.

Drug-resistant TB notification was not included in this study because surveillance systems and practices are distinct and treatment provision and treatment are more carefully regulated; thus, misreporting is minimal.

### Data Collection

For this study’s purposes, the numerator (notified TB) was considered the sum of the cases recorded in the LGA TB notification registers because they contained case-based data.

The LGA registers of notified TB were digitized by trained data entry clerks before fieldwork. These complete case-based notifications were preloaded on the tablets used by data collectors to preclude full-field transcription of patient registers in engaged facilities with a high previsit probability of notification, which was deemed too time-consuming and error-prone; therefore, a hybrid strategy of high-specificity verification was developed and tested.

Data collection instruments were developed, pretested, piloted, and redesigned to reduce respondent burden and focus on variables essential to record linkage. Data dictionaries in the web-based platform were aligned with variables in the national R+R tools. Data on TB cases treated in 2015 were collected from mid-June 2017 to September 2017. Case notification is a human behavior, and the Lagos hybrid method employed HF strata-specific recruitment and data collection approaches in recognition of the different norms, concerns, and motives of each HF stratum.

#### Data Collection in Engaged HFs

In addition to seeking TB cases in all departments of each facility, data officers verified the existence of each previously notified TB case (see Textbox 2 for key case variables). For a *definitive match* (verification), the data officers ensured that the tablet and register matched exactly the following 7 variables: first name, last name, age, gender, date treatment started, smear status, and treatment outcome. A strict deterministic 7-variable match algorithm was chosen to maximize the specificity for linkages. If there was a single discrepancy between a source document and notified case on any of the 7 variables, the data officer deemed it a *nonmatch* and entered the data of patients with TB as a possible *unnotified TB case* to be further scrutinized by probabilistic methods.

**Key case variables.**

- First name
- Surname
- Address (ward and state)
- Date of diagnosis
- Age
- Sex
- Smear done
- Chest x-ray done
- Tuberculosis type (smear result and GeneXpert mycobacterium tuberculosis and rifampicin resistance result)
- Tuberculosis location (pulmonary vs extrapulmonary)
- Treatment outcome
- TB and leprosy supervisor assigned identification of patient (if applicable)
- Facility code

#### Data Collection in Unengaged HFs

The quality and completeness of records in the unengaged sector are known to be highly variable, and a highly adaptive, responsive data collection procedure was needed to gather information. A retrospective approach relies on the existence of patient records in some form. Earlier studies have shown that only 66% of private providers reported having formal patient record systems that summarized the care rendered [21]. Patient information was stored in exercise books (52%), national registers (29%), or forms conceived by the facility staff (15%) [21].

Data collection at unengaged facilities was different from engaged HFs in 3 respects: (1) an incentive (US $5) was offered to private unengaged HFs for participation; (2) a smaller set of variables was collected to reduce privacy concerns (eg, no HIV data); and (3) given the absence of TB registers, data officers had to search for any available patient or diagnostic primary sources and abstract those TB variables that were possible to abstract in an opportunistic way.

We anticipated high rates of refusal (and/or delayed acceptance) among private unengaged HFs because of name-based data collection requiring disclosure of privileged personal information on clients. We sought to reduce this risk of bias by inviting private providers to informational events where influential stakeholders could dispel rumors and encourage participation. We also sought to give facility managers choices over how, how much, and when data were collected, including the option of partial data provision.

Structured interviews with health care providers were conducted to explore self-reported notification knowledge, attitudes, behaviors, and support. Health care workers not currently reporting TB cases were invited to indicate how they would respond to the menu of incentives and enablers to notify TB cases. The responses were written on paper instruments by trained data collectors. Information on the structural-, facility-, and provider-level barriers to notification and reasons for underreporting, nonreporting, and overreporting were collected.

A focus group discussion (FGD) of TB and leprosy supervisor was conducted by an experienced facilitator using an FGD guide in a mix of English and Yoruba. A transcript of the audio recording was produced, and framework analysis was used to derive key challenges and recommendations [22].

### Ethical Considerations

The study required collecting demographic information for the purposes of record linkage (eg, date, age, sex, first name, and last name). Therefore, strict data security procedures were followed to prevent the inadvertent or deductive disclosure of the identities of TB clients and patients, health workers, and facility staff. Data were encrypted and password protected. No provider names were collected to prevent inadvertent disclosure of the identities of health care providers who are in violation of the obligation to notify TB cases; facilities were only identified using numerical codes. All investigators completed the Nigerian research ethics certification courses. The complete protocol was approved by the Health Research and Ethics Committee of the Lagos State University Teaching Hospital (registration number 04/04/2008).

### Data Management

Surveillance data on TB case notifications were single entered into secure relational databases programmed in MySQL following standard operating procedures (SOPs). Fields contained both hard and soft edits, with automated logic checks to increase data quality. During the study implementation, 14 facilities were visited by 2 independent teams to assess the study procedures’ inter-rater reliability for finding cases. Access to the data dashboard was limited to investigators and staff, and web-based access to raw data was limited to those with administrator privileges.

For all patient data collected at any site, the study software automatically generated an alphanumeric case ID code using first name, last name, age, and sex. Given the ethnic diversity in Lagos, the variance in possible first and last names is higher than in homogeneous settings. This greatly enhances the efficiency of record linkage via name-based matching.

### Data Analysis

**Figure 4:**
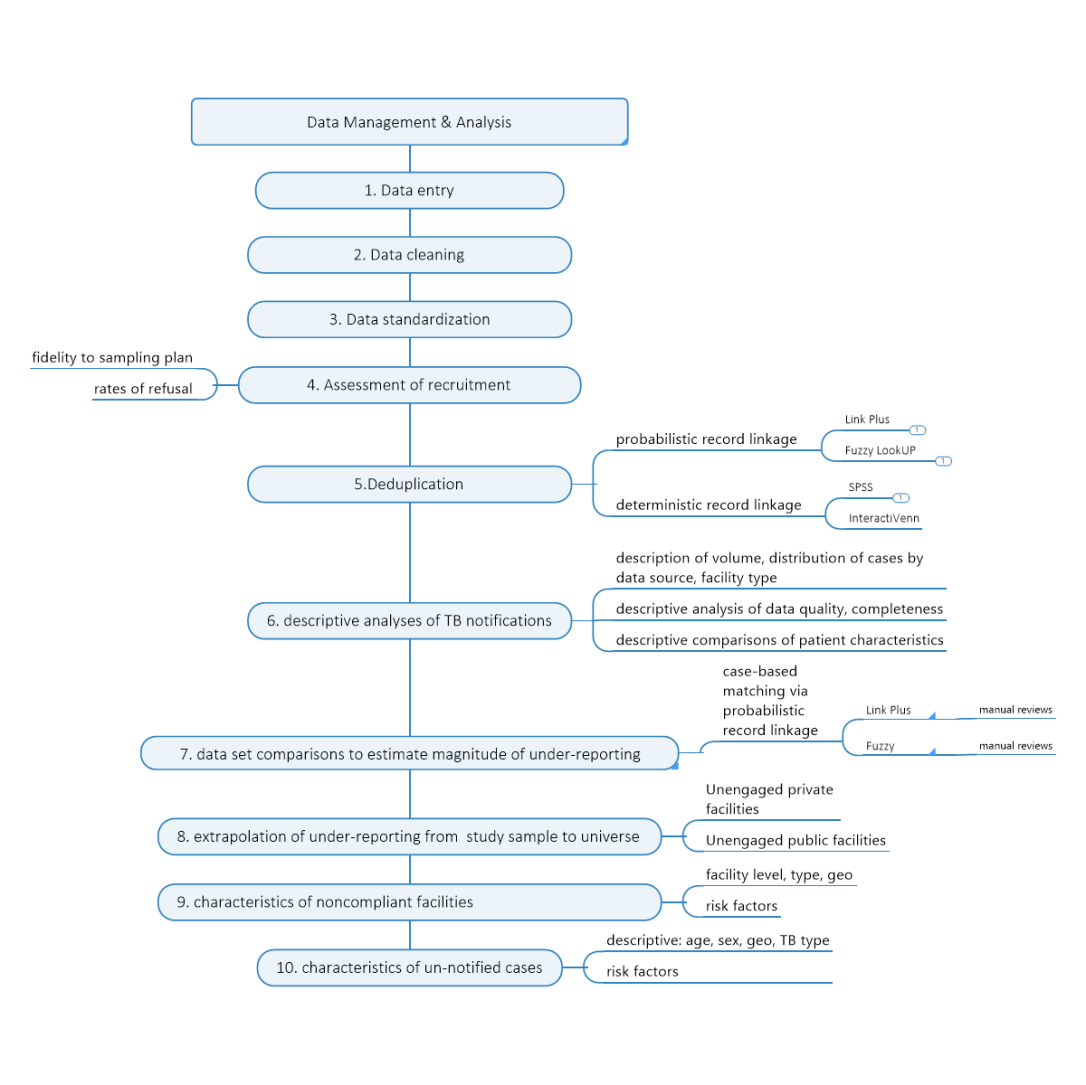
Overview of the sequence of data management and analysis steps. TB: tuberculosis.

As the magnitude estimates are highly sensitive to the bias in sampling, participation, and data quality, transparency in data management is crucial. Figure 4 summarizes the order of the tasks.

An inventory of all information collected from any source was compiled. Missing files, consents, or duplicate facility visits were investigated by the data manager. Data were cleaned and validated by the data manager and the principal investigators in January 2018. An audit trail was maintained to indicate changes to the data sets during cleaning.

In this study, the identification and removal of duplicates through record linkage was a precursor for comparison between sources. Probabilistic record linkages were conducted to deduplicate the data sets (Multimedia Appendix 2 [11,18]).

#### Study Objective 1: Number and Proportion of TB Cases Treated

The calculation of the sum of all unique TB cases diagnosed in Lagos in 2015 (the denominator) required extensive record linkage to avoid double counting of patients with TB appearing in multiple data sets. The engaged facility register data were compared with the LGA register data using probabilistic record linkage to identify TB cases that had not been notified.

##### Estimation of Total Unnotified Cases From Facility Samples

The proportion of unnotified TB cases in each sampled stratum was extrapolated to construct the total number of unreported cases. The record linkage of B+ TB cases diagnosed in laboratories was compared with B+ patients in different databases to estimate the number of B+ patients with TB who were treated in the unengaged sectors or were untreated.

As Nigeria does not have a system of unique personal identifiers for TB, probabilistic record linkage for smear and GeneXpert positive TB cases was essential to compare laboratory and facility records to establish the number of B+ TB cases treated in the unengaged sector (or either untreated). The comparison of laboratory and register cases was conducted with Excel Fuzzy LookUp and CDC LinkPlus because of the limited number of potential record linkage variables and the ability of Fuzzy LookUp to compare information across variables instead of only between variables. LinkPlus failed to identify matches when the order of names was reversed, whereas Fuzzy LookUp could consider similarities in a group of variables (eg, first and last names jointly; Multimedia Appendix 3).

##### Extrapolation of Total Patients With TB Volume From the Unengaged Private Sample

To calculate the size of the population of patients with TB treated in the unengaged sector, 2 approaches were used. To estimate the total number of B+ patients with TB treated outside the engaged HF, a direct measure of total B+ TB diagnoses was feasible because of the inclusion of all laboratories in the state. The number of B+ patients with TB treated in the unengaged private sector was presumed to be the total number of B+ patient test results remaining after removing samples linked to treated patients with TB in TB treatment registers (accounting for cross-border diagnoses). This was a reasonable approach because the study was effectively a census of all known laboratories in Lagos, and therefore, it captured the universe of samples testing positive for TB.

**Figure 5:**
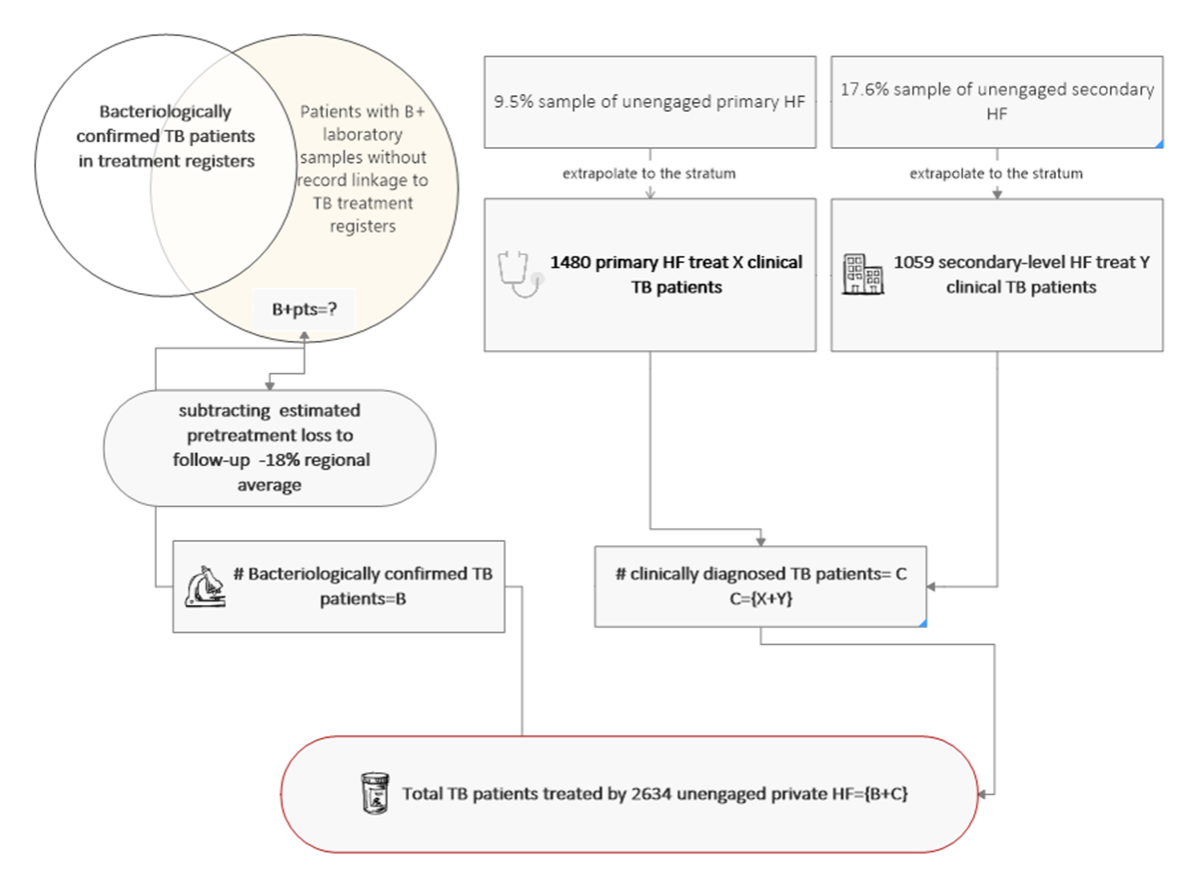
Process used to estimate the total number of tuberculosis cases treated in the unengaged private sector. B+: bacteriologically confirmed tuberculosis; HF: health facility; TB: tuberculosis.

To calculate the number of clinically diagnosed patients with TB treated by the unengaged private sector, we took the number of unique clinically diagnosed patients with TB resident in Lagos found by each stratum (level) of the facility and produced weighted strata-specific averages that were then applied to the universe and summed to obtain the total. Adding the total B+ cases and the clinically diagnosed cases together yielded the estimate of total TB treated by the unengaged private sector (Figure 5).

As pretreatment loss to follow-up could not be measured in the HFs that were not sampled, we assumed the continental average pretreatment loss to follow-up applied to Lagos residents testing positive for TB. A systematic review found pretreatment loss to follow-up averages of 18% (95% CI 13-22%) in Africa [23].

**Figure 6:**
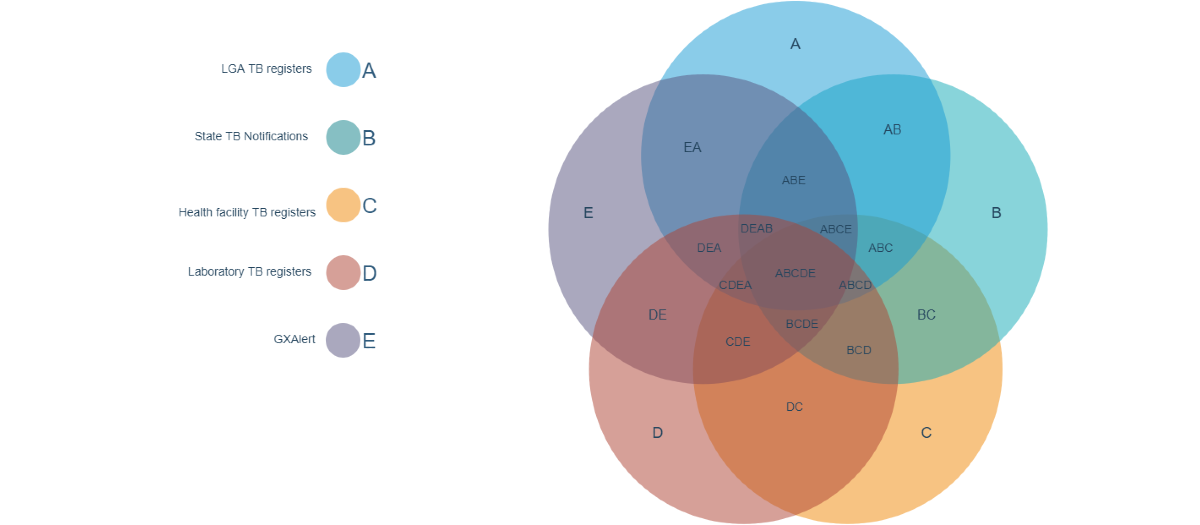
Comparison of TB data sets to discern the level of misreporting of TB notifications. LGA: local government area; TB: tuberculosis.

In addition to assessing undernotification, this inventory study included a comparison of the volume and characteristics of TB cases in 3 layers of the TB surveillance system. For comparisons of 3 or fewer data sets, Euler and Venn diagrams were generated to maintain proportionality. EulerAPE v3 software was used for area-accurate proportional Euler diagrams [25]. For comparisons of 4 or more data sets, spherical or symmetrical Venn diagrams were necessary. Interactivenn and Jvenn were used for spherical or Edwards-Venn diagrams (Figure 6) [26,27].

##### Adjusting for Cross-Border Diagnosis and Treatment

The WHO inventory study methods may assume no health care provision across geographic borders, an unlikely situation. Due to the elongated shape and riverine coastal areas of Lagos, it is often more convenient to cross the border into Ogun than to traverse Lagos for health care. To account for cross-border care seeking in estimates of undernotification, it was necessary to measure the magnitude of the cross-border diagnosis and treatment phenomena between Lagos and Ogun. We conducted probabilistic record linkage among TB treatment registers in 3 contiguous LGA in Ogun (bordering Lagos) and smear-positive lab samples in Lagos, and a buffer zone analysis was conducted to estimate the magnitude of misreporting because of cross-border care seeking.

#### Study Objective 2: Facility Compliance With Obligations to Notify

As the LGA register and facility register data were case-based, these data permitted facility-level analyses following aggregation of the data. Syntax was written for analyses using the SPSS (IBM version 25). The number, proportion, and type of TB facilities that did not comply with the obligation to notify in 2015 (public and private) were calculated to help pinpoint areas for intervention.

We characterized the engaged facilities as *active*, *semidormant*, or *dormant*, based on whether they were providing TB treatment in 2015. HFs that served an average of less than 1 patient per month were considered *semidormant*. Active and semidormant HFs were included in an analysis of the completeness of notifications. They were subsequently *compliant* or *noncompliant* with reporting requirements. Categorization of *noncompliant* HFs includes accurate reporting (ie, zero difference between facility and LGA register), underreporting (ie, reporting fewer cases than patients recorded in the treatment register), and overreporting (ie, reporting cases that could not be verified in the facility register). Multiple imputations were conducted to estimate the magnitude of misreporting because of refusals among eligible HFs.

#### Study Objective 3: Comparison of Characteristics of Notified and Unnotified Patients With TB

Beyond the estimation of the magnitude of underreporting, it is necessary to understand the differences between TB cases reported and TB cases unreported. Differences may include variation in the quality of diagnosis, treatment, patient population served, geographic area, and types of facilities that opt to notify TB and those who do not. Descriptive statistics of notified TB cases (eg, LGA distribution, types of TB age, gender, treatment outcomes, and source of notifications [facility type]) were generated to compare service delivery by sector.

TB case characteristics in each data set were described and compared between and among data sets to discern if there were any differences in the type of people or type of TB treated in the various sectors. The magnitude and variance of the cases reported were examined to identify the central tendencies and outliers. Percentages and means were calculated for numerical variables; the chi-square test or Fisher exact test was used to compare categorical variables, whereas two-tailed t tests were used to compare continuous variables. For all statistical tests, *P*<.05 was considered statistically significant.

To discern the implications of underreporting, we conducted descriptive analyses to detect differences among notification systems, facilities, and cases. Data were analyzed in a stepwise fashion according to descending aggregation levels: LGA, facility, and person. The movement of B+ patients within and between HF types was mapped to explore retention, referral, and treatment choices.

#### Study Objective 4: TB Surveillance Coverage and Quality

We compared participation levels in the 2 state-run disease surveillance systems in terms of TB to understand their geographical scope and structural differences and make inferences about the quality and reliability of the estimates generated.

#### Study Objectives 5 and 6: Barriers to Notification, Reasons for Misreporting, and Potential Solutions

To derive actionable recommendations to improve notification and surveillance quality at the state, LGA, and facility levels, we analyzed each survey separately. We then triangulated the results of all the data sets (eg, health care worker survey, registers, and FGDs) to assess discrepancies between self-reports and facility-level reports. Syntax was written for analyses using the SPSS IBM version 25. Health care worker interview data were appended to the corresponding facility visit report. The magnitude and variance of the responses were examined to identify central tendencies, and outliers were considered for further exploration. Percentages, means, medians, and interquartile ranges were generated to describe survey samples. The chi-square test or Fisher exact test was used to compare categorical variables; *t* tests were used to compare mean values between groups with parametric continuous variables (eg, trust scores and stigma scores). *P*<.05 was considered statistically significant for all statistical tests, and 95% confidence intervals were generated for all point estimates. Odds ratios (ORs) were generated to explore associations. Scales were assessed for reliability with Cronbach alpha, and items with poor performance were deleted.

The focus group transcripts with the local government TB and leprosy supervisor on challenges with the surveillance system were analyzed using the framework method and summative content analysis by a multidisciplinary team with knowledge of Nigerian and Lagosian culture and languages [22].

## Results

### Pilot

The first pilot suggested that many study procedures and instruments were overly complex and insufficiently clear for data collectors and respondents. A thorough redesign was necessary. The verification of cases in DOTS facilities yielded 100% verification, suggesting low sensitivity and specificity to discrepant information. Instruments, procedures, training, SOPs, supervision, and community entries were revised internet field challenges, obliging a paper-based back-up system and multiple internet connectivity options. Instruments and data collection forms were shortened to improve data quality, reduce respondent burden, and enhance respondent assent. An unacceptable rate of participation (0%) among unengaged private facilities in the pilot obliged the addition of a small monetary incentive (US $5).

In the second pilot study, the revised SOPs, shortened instruments, and retrained data officers were tested. Although fewer challenges were identified, the web app was updated to correct misclassifications, misspellings, and validation rules. Questionnaires had a lower respondent burden, and the time per facility was reduced to acceptable levels.

### Study Results

As the survey was implemented, participation was robust, and survey implementation proceeded per protocol. In the original population proportional to size sample, a total of 605 HFs and 349 laboratories were included. Recruitment followed the per protocol sampling plan in terms of facility type and facility level, but geographical distribution varied in laboratories because of the high frequency of substitution owing to ineligibility. Ultimately, via sampling with replacement, 608 HFs (100%) and 328 (93.9%) laboratories participated. Public unengaged facilities that were purposefully selected because of ambiguity regarding their engagement status were less likely to participate (6/23, 26% refusal); 1 in 10 unengaged private HFs approached was out of business (45/381,11.8%) or was unwilling to participate (36/381, 9.4%). An unengaged private HF that was unwilling to participate was replaced with an HF similar in level and LGA. Refusal and proportion granting partial access to patient records (15/381, 3.9%) among unengaged HFs were lower than anticipated, suggesting that selection bias was minimized. The use of a monetary incentive in this stratum may have mitigated the risk of bias of refusal.

Among the 608 facilities enrolled in the study, 564 (92.7%) granted full access to all facility records and 44 (7.3%) granted partial access to facility records. Health care worker survey refusal was moderate (13%).

In *dormant* facilities, there were often no eligible respondents; 3 of the 5 tertiary level public institutions refused to participate in the health care worker survey, which reduced the representativeness of this specific stratum, which is prone to misreporting.

As it was not feasible to include all 2561 unengaged HFs in Lagos, our misreporting estimates are extrapolated from a sample. The sample estimates (7%- 10%) case notification in the unengaged private HFs were below the 3% notification observed. Moreover, the estimates of the frequency of TB treatment provision (32%-26%) were triple the frequency (11.4%) observed in the survey. Valid generalization from a sample to a population relies on a solid understanding of the heterogeneity of the sites and strict fidelity to sampling with replacement per protocol. Knowledge of all types of heterogeneity and variance among unengaged sites was incomplete because of the dynamism of this sector. HFs frequently changed ownership, location, name, services, and level, and although a valid sampling frame was assembled in 2016, changes occurred in the 9 months between the generation of the frame and when data were collected.

### Number and Proportion of TB Cases Treated

The calculation of the total volume of TB treated in the unengaged private sector relies on 2 specific assumptions: (1) that B+ TB samples that could not be linked were treated by unengaged private providers and (2) that pretreatment loss to follow-up occurs at the average rate. The use of these parameters allowed us to overcome potential bias concerning record decay at the facilities, but it also introduced a degree of uncertainty into the estimate. Partial access to patient records in 3% (3/98) of DOTs facilities may have also affected our estimate of TB case undernotification. However, as this affected only 3 sites, we decided to adjust for it.

Table 2 summarizes the performance of the Lagos hybrid methodology as compared with the WHO inventory methods.

**Table 2:** Findings and lessons derived from the implementation of the Lagos hybrid inventory methodology.

WHO^a^ inventory methods	Lagos hybrid inventory methodology	Findings and lessons
Electronic case-based national TB^b^ surveillance system	Retrospective digitalization of paper-based surveillance to create electronic case-based surveillance system	Quality-assured digitalization requires robust database design and data management. Relational databases are required where each element has a unique ID, including each facility.
Electronic case-based database with records for patients with TB	Digitalization of facility and laboratory records with no notification behavior	The inability to distinguish between sites that diagnosed no patients with TB and those who kept no records of patients with TB treated is a weakness of retrospective designs. The decay of records was an issue. Some patients’ paper records were physically damaged in 5 out of 701 sites through poor warehousing or force majeure, impacting the quality of both verification and case finding. As 4 of these sites were engaged, this may have led to the underverification of notified cases, leading to an inflated estimate of overreporting. This can be mitigated by triangulation and minimizing the period between reference year (2015) and data collection (2017).
Electronic case-based database with records for patients with TB	High-specificity on-site verification in engaged HFs^c^ with the likelihood of notification behavior	On-site human verification of notified cases using a deterministic 7 variable matching algorithm in engaged DOTS^d^ centers with a high likelihood of notification proved a viable alternative to 100% field-based data entry. It was acceptable to providers because TB registers did not need to be removed from the premises, on-site data entry was minimized, and the total respondent burden was reduced.
Census of all HF (retrospective) or random sampling of all HF (prospective)	Hybrid mix of census and stratified probability proportional to size sampling methods among HF strata	The census of laboratories paired with the sampling of private HFs was robust because it allowed for triangulation of self-report, as well as extrapolation. However, the frequency of TB service provision (32%-36%) in the unengaged private HFs was significantly overestimated, so the point estimate of misreporting of clinical TB in the unengaged private sector has large uncertainty bounds. This large sampling error would have compromised the estimate of misreporting of B+^e^ TB also, were it not for the ability to rely upon the TB diagnoses from a census of all laboratories.
Standardized TB case definitions	Broad case definition for TB (all other forms); standardized case definition for B+ TB	The use of a broader definition of clinical TB permitted a more complete accounting of TB treatment coverage that includes overdiagnosis and overtreatment. Documentation of the frequency of diagnosis of TB without bacteriological testing in the unengaged sector is important information for public health stakeholders. The clinical diagnosis was a very small proportion of TB treatment found (11%).
Presence of unique identifiers for record linkage for deterministic record linkage	Use of multi-variable probabilistic record linkage algorithms with sensitivity analysis, combined with an independent review	Probabilistic record linkage with WHO-recommended software underestimated notification due to low sensitivity for name reversal. Use of Excel add-in (Fuzzy Lookup) allowed for matching across and between variables but is not syntax driven and provides no audit trail. The ability to account for name order reversal is important to avoid bias in misreporting estimates.
National in scope or sampled “self-contained” geographical areas	Subnational in scope, but using buffer zone sensitivity analysis to permit estimation and adjustment for cross-border health care seeking	Buffer zone sensitivity analysis is straightforward to conduct and permits robust subnational and urban inventory studies.
No recommendation to study misreport between levels of the TB surveillance system	Comparisons of aggregated data to identify misreporting between administrative levels of the notification system	The addition of within-surveillance system misreporting enhanced the value of the study for local stakeholders.
No recommendation to include the study of the underlying rationales and solutions to suboptimal notification behaviors in addition to its quantification	In-depth interviews with health care providers and focus group discussions with surveillance offers were undertaken	Focus group discussions and in-depth interviews provided context for quantitative findings of misreporting, critical to engage stakeholders in identifying roots of and solutions to the notification problem.

^a^WHO: World Health Organization.

^b^TB: tuberculosis.

^c^HF: health facility.

^d^DOTS: Directly Observed Treatment Short-Course.

^e^B+: bacteriologically confirmed tuberculosis

### Adjusting for Cross-Border Diagnosis and Treatment: Buffer Zone Sensitivity Analysis

**Figure 7:**
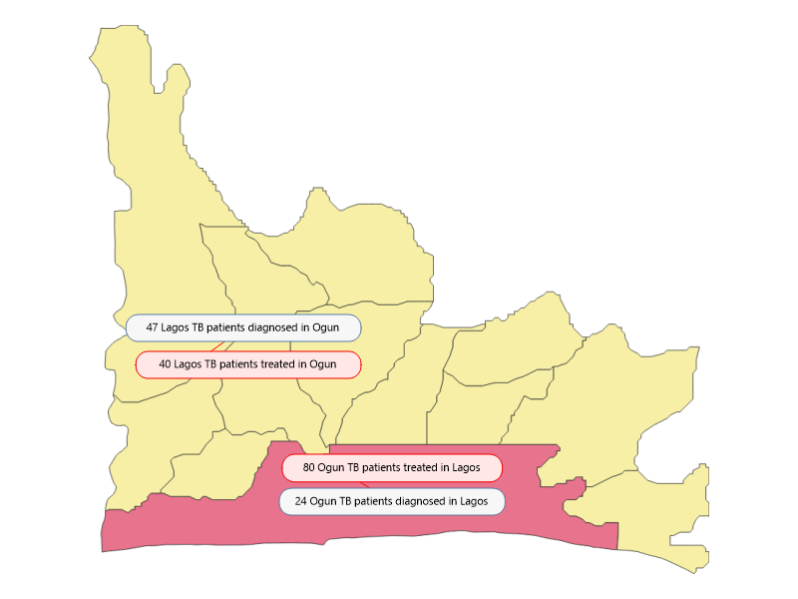
Schematic of cross-border TB diagnosis and treatment. TB: tuberculosis.

In the Ogun laboratory data, 47 Lagos residents were diagnosed, and in the sampled HFs, 80 were treated (see Figure 7). Residents with Ogun addresses in the Lagos TB treatment registers were also identified. The results suggested a low level of cross-border care seeking, with slightly more Lagos residents being diagnosed in Ogun and slightly more Ogun residents seeking treatment in Lagos. The absolute numbers of cross-border TB treatments were deemed so small, so as to have minimal influence on the analysis.

### Comparison of Characteristics of Patients With TB Notified and Unnotified

Patients with TB who were never notified to the program had similar age and sex distribution to those who were notified. However, they differed in other aspects. Individuals with unknown treatment status were more likely to have had their TB diagnosed via the GeneXpert test than with smear microscopy (19% vs 12%; OR 0.60, 95% CI 0.52-0.71). This suggests that persons living with HIV may be less likely to start treatment for TB and/or that pretreatment loss to follow-up was higher in sites equipped with GeneXpert mycobacterium tuberculosis and rifampicin resistance machines. Treatment success was modest in all sectors (*R*=37.9%-72.0%) and did not align with treatment success reported by the state (6665/8770, 75.99%).

### TB Surveillance Coverage and Quality

We obtained aggregated LGA level monthly TB totals of inpatients and outpatients from the IDSR office. We sought IDSR facility total monthly TB notification data, but we were unable to obtain them from LGA officers during the course of the study or during their supervision meeting, which limited the granularity of the analyses. As the inpatient and outpatient department values contain duplicates and therefore cannot be directly compared with LGA, notification comparisons were of limited value. As the IDSR and STBLCP data were aggregated, it was not possible to reconcile them fully. Descriptive analyses are included in Multimedia Appendix 4.

### Barriers to Notification, Reasons for Misreporting, and Potential Solutions

Notification was rarely a top priority for TB care providers. When asked what might be done to improve the TB notification system, some instead advocated for regular drug supplies; GeneXpert machines; laboratory personnel; health education materials for patients; more clinical and laboratory staff; and greater efforts at community mobilization, early detection, and financial support for patients with TB. Among those who identified reporting as uncomfortable, the reasons for discomfort with disease notification included practical, logical, strategic, and economic concerns. The most common reason given by both TB providers and nonproviders was the lack of access to notification forms and registers. A quarter of TB providers (26%) were doubtful that the volume of patients with TB they treated in their facility merited mastery of the extensive TB notification forms and procedures.

## Discussion

The WHO strongly recommends that high-burden countries undertake measurement of underreporting while simultaneously setting the methodological bar so high, so as to preclude most TB programs in high-burden settings from attempting one [4]. This study attempted to design an inventory study that struck a balance among competing demands for rigor, ethics, efficiency, and feasibility for high-burden settings with a lack of standardized case definitions; electronic case-based surveillance infrastructure; or closed catchment areas. Combining elements of WHO-endorsed study designs and improvising techniques to address validity threats allowed this study to capture the level of misreporting in a high-need setting. The use of the hybrid method led to robust participation.

Solid participation of HFs may be a consequence of minimizing respondent burden, iterative piloting, and incentives for unengaged private HFs. Incentives for health care workers who work in high refusal strata (eg, tertiary care public HF) should also be considered.

Although the methodology proved robust and responsive to stakeholder needs, multiple methodological trade-offs should be borne in mind by researchers undertaking the hybrid method. First, the inability to distinguish between HF that diagnosed no patients with TB and those who did not have records of patients with TB treated was a shortcoming of the chosen retrospective design. This flaw was offset by the census of all laboratories and could be used as the basis for B+ TB caseloads. However, for clinically diagnosed cases, zero reports could not be verified. Relatedly, the retrospective design was susceptible to ascertainment bias. It is crucial to collect data as immediately after the reference period as possible, as the physical decay of paper records is a threat. The existence of written records for consultation varied widely and is likely correlated with notification behavior and nonrandom bias that lab data triangulation overcame.

Although sampling assumptions of underreporting (90%-93%) proved an underestimate (97%), the frequency of TB treatment in the unengaged private HFs was overestimated, so the sample size was adequate. The inclusion criteria for private unengaged HFs did not include providers such as patient medicine vendors, corporations offering occupational health services, or mobile clinics. These providers were not thought to diagnose or treat TB in 2015. However, community screening plays a very important role in TB diagnosis in many urban areas, including Lagos, in 2020, and exclusion of these entities should be considered.

One of the strengths of this methodology over the WHO-recommended approaches is that it frames TB case notification as an institutional behavior influenced by sector-specific motivations, policies, politics, infrastructure, and challenges. Using HF strata as sampling units leverages social scientific research and stakeholders' knowledge of notification attitudes and practices. Our methodological use of prior knowledge of notification behaviors reflects a growing call to use Bayesian approaches in inventory studies [28]. In contrast, the WHO inventory study methods draw upon wildlife ecology, assuming random movement of cases and making no attempt to gather data on motives, interests, or abilities of persons working in HFs. One WHO method (capture-recapture) even assumes that TB cases have an equal probability of notification (homogeneity capture), a stance that belies the different stakes providers have to engage in public health surveillance. Framing TB notification as a necessary but labor-intensive, poorly incentivized choice allows researchers to shift the measurement focus from mere counting cases to understanding the knowledge, behavior, and needs of gatekeepers. Our results confirm that many TB providers considered notification an additional burden with unclear utility.

Although our study methods are likely to be applicable to other urban areas, comprehensive, iterative piloting is needed for replication. The probabilistic record linkage possible in Lagos is unlikely to be appropriate for more ethnically homogeneous areas with lower levels of diversity in first and last names.

We show that in settings with weak surveillance systems, hybrid approaches may even be preferable to designs that assume notification is random and oblige significant intervention at HFs because the Lagos hybrid method does not distort *natural* R+R behaviors, is acceptable, and is feasible in settings where inventory studies are most needed.

### Conclusions

This study adapted the WHO undernotification estimation methodology and procedures to take into account known surveillance system strengths, weaknesses, seasonality, and (crucially) local stakeholders’ needs for actionable information on the causes of TB misreporting. Participatory and locally tailored methods met policy makers’ needs for causal information and feasible recommendations. Subnational inventory studies in high-burden settings with weak paper-based surveillance, dynamic private sectors, and open borders are both feasible and informative. The WHO preconditions should not discourage high-burden countries from measuring misreporting as part of the process of solving notification challenges.

## Appendix

Multimedia Appendix 1 Sampling frame deduplification methods and outcomes.

Multimedia Appendix 2 Sampling plan.

Multimedia Appendix 3 Standard operating procedures.

Multimedia Appendix 4 Descriptive analysis of TB surveillance in Lagos, 2015.

## Abbreviations

B+: bacteriologically confirmed tuberculosis
CDR: case detection rate
DOTS: Directly Observed Treatment Short-Course
FGD: focus group discussions
HEFAMAA: Health Facility Monitoring and Accreditation Agency
HF: health facility
IDSR: Integrated Disease Surveillance and Response
KNCV: Koninklijke Centrale Vereniging tot bestrijding der Tuberculose
LGA: local government area
NTP: national tuberculosis program
OR: odds ratio
PPM: public-private mix
R+R: recording and reporting
SOP: standard operating procedure
STBLCP: State Tuberculosis and Leprosy Control Program
TB: tuberculosis
USAID: United States Agency for International Development
WHO: World Health Organization
