# Supplementary material for: Hybrid Approach to Estimation of Underreporting of Tuberculosis Case Notification in High-Burden Settings With Weak Surveillance Infrastructure: Design and Implementation of an Inventory Study
Source: JMIR Public Health Surveill. 2021 Mar 15;7(3):e22352. doi: 10.2196/22352 (PMC8088841; doi:10.2196/22352)
Supplement: Multimedia Appendix 1 [file publichealth_v7i3e22352_app1.docx]

Supplemental File 2: De-duplification

Double counting may occur in the following circumstances:

1. Patients initially managed as in-patients in tertiary and secondary health facilities are later referred to a DOTS center for commencement of TB treatment when stabilized.
2. Patients diagnosed at a DOTS facility are referred to another DOTS facility to commence TB treatment because it is closer to their residence.
3. Patients diagnosed at unengaged public or public and private health facilities and referred to a DOTS facility to commence treatment because treatment is not offered at diagnostic site
4. Patients initially managed at unengaged public and private health facilities are either referred or decided on their own to get treated at a DOTS facility because cost of treatment is too high over the term.
5. Patients commenced on drug-sensitive TB regimen, but later switched to MDR regimen.

Each approach and software has strengths and weaknesses and it was therefore prudent to employ different strategies depending on the number of variables for matching, the need for precision, ease of use, and the data quality.[21, 22]

Table S2-1: comparison of features of software for record linkage

| Record linkage tool | Match Type | strength | weakness |
| --- | --- | --- | --- |
| CDC LINK plus | probabilistic | Built for further manual classification | Can only link databases of fewer than 25 variables, does not automatically create a matched set. |
| Excel Fuzzy LookUP | probabilistic | Allows matching across variables (good for name reversal), automatically joins matched data | Requires large computing memory RAM to run |
| SPSS | deterministic |  | Low sensitivity by definition (underestimates matches) |
| InteractiVenn[23] | deterministic | Web-based, easy to make spherical Venn with up to 6 datasets | Under estimates matches, case sensitive |
| Jvenn[24] | deterministic | Web-based, easy to download sets of data | Under estimates matches, case sensitive |

To estimate the number of unique TB cases in the LGA dataset of “Notified TB cases” (n=8,702), a variety of de-duplification strategies were employed. It was not possible to establish variable weighing a priori due to insufficient prior insight into the relative uniqueness of each variable. In theory variables with more characters would seem to be more unique and thus more valuable for record linkage, but they also tend to have more random error (typos) that reduce the value. So we were agnostic toward the value of specific variable for record linkage, preferring to run five record linkage models for de-duplification of case-based data (See probabilistic linkage models in Appendix 3). Linkage methods included deterministic and probabilistic approaches.

The record linkage algorithm with the highest number of probable record linkages was selected for manual review by two independent raters. Manual reviews were necessary to examine the possibility of:

1. duplicates to be retreatment of the same individual (examine of date of treatment start and treatment outcomes
2. double counting of patients who transferred

The result was 8,436 unique records of TB patients (97%: 8,436/8,702). De-duplification of the LGA dataset of “Notified TB cases” also provided an estimate of the magnitude of “over-notification” also referred to as “over-reporting”.

## De-duplification models for LGA Register data

To estimate the number of unique TB cases in the LGA dataset of “Notified TB cases” (n=8,702), a variety of de-duplification strategies were employed. It was not possible to establish variable weighing a priori due to insufficient prior insight into the relative uniqueness of each variable. In theory variables with more characters would seem to be more unique and thus more valuable for record linkage, but they also tend to have more random error (typos) that reduce the value. So we were agnostic toward the value of specific variable for record linkage, preferring to run five record linkage models for de-duplification of case-based data (See probabilistic linkage models in Appendix 3). Linkage methods included deterministic and probabilistic approaches. The linkage with the highest number of potential matches was selected for manual review by two independent raters. De-duplification of the LGA dataset of “Notified TB cases” also provided an estimate of the magnitude of “over-notification” also referred to as “over-reporting”.

We ran 5 linkage models for de-duplification of case-based data in the LGA registers. Linkage methods included deterministic and probabilistic approaches. The linkage with the highest number of potential matches was selected for manual review by two independent raters. The result was 8,436 unique records.

Algorithm 1 gives additional weight to the name, age and sex variables by counting them twice (within CASE ID. It also treats age as relative which allows for aging. Algorithm 2 uses fewer variables which in and of itself reduces the likelihood score and lowers the match potential. By removing CASE ID, it doesn’t doublecount names. Removal of treatment start date as a match criterion makes it easier to pick up matches across facilities, where patients are shopping for treatment or there are relapses. Adding back in case ID in method 5 increases the volume of matches. First we reviewed the name-based dataset from the TBLS (LGA-level). The result was 8,436 unique records (97%: 8,436/8,702).

#### Table 50: De-duplification models and yields for LGA case-based TB register data

| **LGA TB registers 2015** | **pairs** |
| --- | --- |
| **Model 1- deterministic record linkage in SPSS- 3 variables**  After we corrected the files to change string variables to all CAPS, We both independently found 94 deterministic matches using SPSS record linkage on first name, last name, and case ID (i.e. Sex and age). | 94 |
| **Model 2- fuzzy Link Plus 5 variables**  we ran a fuzzy match in Link Plus record linkage on first, last, case ID, address, and age – with a cut-off value of 7.0 yields a range of probability scores between 7.1-34.4 | 276 |
| **Model 3- fuzzy Link Plus 7 variables**   - Blocking variable: disease site - Fuzzy matches: first name, last name – treated as generic string variables - Age – generic string - Sex – exact match - Address – fuzzy match - CASE ID - Data of Treatment start – treated as a date - A cut-off value of 8 was employed. | 266 |
| **Method 4 – fuzzy Link Plus 5 variables**   - Blocking variable: disease site - Fuzzy matches: first name, last name – treated as generic string variables - Age – exact match - Sex – exact match - Address – fuzzy match - A cut-off value of 8 was employed. | 178 |
| **Method 5 – fuzzy Link Plus variables 6**   - Blocking variable: disease site - Fuzzy matches: first name, last name – treated as generic string variables - Age – exact match - Sex – exact match - Address – fuzzy match - Case ID – fuzzy match - A cut-off value of 8 was employed. | 187 |

## De-duplification models for engaged DOTS facility TB register data

The high specificity verification process involving deterministic matching on 7 variables is intended to produce duplicates. The data entry SOP employed at the engaged facilities that mandated any slight discrepancies to be considered non-matches and patients were entered as ‘new records’, as described in the verification methods section. To identify and remove these duplicates from the denominator, both probabilistic and deterministic record linkage models were employed.

There were 11,531 TB case records entered by data collectors at engaged facilities, including the pre-loaded records abstracted from the LGA register. A total of 2,414 pairs (possible duplicates) were identified in Link Plus using the (below) record linkage parameters when the direct method was employed, using a cutoff value of 7.0, and the field for blocking was *Disease Site*. EMHM and OA independently reviewed the 2,414 pairs, and found 2,227 true duplicates, reducing the sum of unique TB cases within the engaged public and private facilities (denominator) to 9,340. Discordance was discussed, and consensus reached. Additional manual cleaning reduced the unique cases further to 9,192.

Record linkage on first, last name, age, and sex, there were 14 possible duplicates in the unengaged new cases (n=160), of which 6 were actual duplicates (and 2 were from a misclassified facility) when all the data were reviewed. So unique unengaged cases fell to 154 in total. These 154 cases were found among 36 facilities in 15 different LGAs.

De-duplification of DOTS centre register data was conducted in Linkplus with independent manual review by 2 investigators. A total of 2,414 pairs were identified in Link Plus using the (below) record linkage model parameters when the direct method was employed, using a cutoff value of 7.0, and the field for blocking was *Disease Site*. EMHM and OA independently reviewed the 2414 pairs, and found 2,227 true duplicates, reducing the sum of unique TB cases within the engaged public and private facilities (denominator) to 9,304. Hand searching identified another set of matches, reducing the total to 9192.

#### Table 51: De-duplification models for engaged DOTS Centre TB registers

| **Model** | **Linking Process** | **Field for Blocking** | **Record linkage Parameters** | **Pairs found** | **True matches following manual reviews** |
| --- | --- | --- | --- | --- | --- |
| 1 | Cutoff Value=7.0 | *Disease Site*. | 1. UPfirstname 2. UPlastname 3. Age 4. Sex 5. UPaddress | 2,414 | 2,227 |
| 2 | Deterministic SPSS | *n/a* | 1. Case ID (first name, last name, age, sex) | 59 | 59 |

After de-duplification with probabilistic methods, a comparison with deterministic methods in SPSS detected 59 duplicates.

## Appendix 5: De-duplification models for laboratory case data

Seventy-six engaged stand-alone and embedded laboratories in the state recorded 5,533 TB-positive samples. A total of 398 individuals appeared twice in the laboratory dataset: 104 (26%) appeared twice because they tested both GeneXpert positive and smear microscopy positive, 51 individuals (13%) had been diagnosed in more than one lab, 45 (12%) were double data entry errors, 27 (6.8%) were follow-up smears that were entered erroneously, and for the remainder (171, 43%) the reason for duplication was repeat testing. After de-duplification, seventy-six engaged labs tested 4,847unique bacteriologically positive TB persons. (See probabilistic linkage models in Appendix 5)

There were no duplicates among the 288 bacteriologically positive persons found in the 276 unengaged labs after deterministic and manual reviews.

De-duplification by Link Plus flagged 526 test results as potential duplicates, and 398 were true matches on manual verification, reducing the number of unique bacteriologically positive labs tests to 5,135. De-duplification was conducted in LinkPlus with manual review. Number of Records = 5,338

#### Table 52: De-duplification models for laboratory data

| **Model** | **Linking Process** | **Field for Blocking** | **Record linkage Parameters** | **Pairs found** | **True matches following manual reviews** |
| --- | --- | --- | --- | --- | --- |
| 1 | Direct method is employed  Cutoff Value=5.0 | LocalGovArea | Record linkage Field   1. PatientFirstName 2. PatientSurName 3. Age 4. TheUniqueIdentifier 5. ReferringHF 6. Laboratory_Name | 526 | 414 |
| 2 | Direct method is employed  Cutoff Value=7.0 | ResidenceState | Record linkage Parameters   1. PatientFirstName 2. PatientSurName 3. TheUniqueIdentifier 4. Sex 5. Age 6. ResidenceStreet 7. LocalGovArea 8. ReferringHF | 526 | 414 |

Using Model 2 and cut-off of 7, a total of 526 potential duplicates were identified in the laboratory data file

Twenty-six duplicates were found via deterministic record linkage, to reduce the unique TB patients in the notification group to 708. There were 22 duplicates via deterministic record linkage in the facility registers, to make 271 unique cases.

Figure 1: Map showing shared border between Ogun (shaded) and Lagos (darker) state


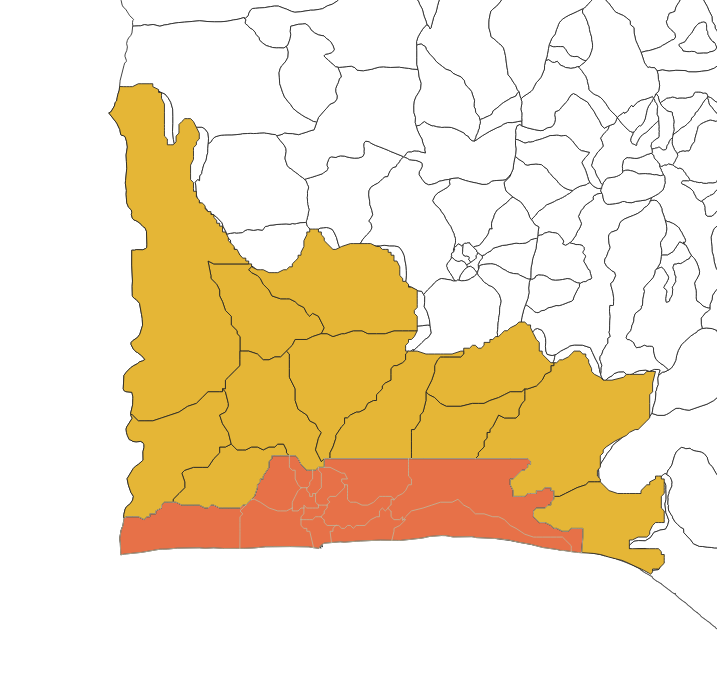


Figure 2: Summary of TB datasets following de-duplification.

* No de-duplication possible as these databases only had aggregate data.
