# Supplementary material for: Hybrid Approach to Estimation of Underreporting of Tuberculosis Case Notification in High-Burden Settings With Weak Surveillance Infrastructure: Design and Implementation of an Inventory Study
Source: JMIR Public Health Surveill. 2021 Mar 15;7(3):e22352. doi: 10.2196/22352 (PMC8088841; doi:10.2196/22352)
Supplement: Multimedia Appendix 2 [file publichealth_v7i3e22352_app2.docx]

## Supplemental File 1: Detailed Sampling Strategies

We anticipated substantial misclassification of TB engagement status because official lists for 2015 did not contain the same elements. Moreover, partners were engaging new facilities and re-awakening dormant DOTS facilities during 2015. Following desk review with the Lagos State TB program team, 315 facilities were determined to be ‘engaged DOTS facilities’ following de-duplification and application of the broadest operational definition:

- 1. Having TB treatment services, and
  2. having received training on TB in the past, or
  3. having received TB drugs from the state program in exchange for information on TB cases treated, or
  4. If private care provider has signed a Memorandum of Understanding (MOU) with the TB program (aka Engagement)

Figure 1 Venn diagram of Samples HF by Engagement status


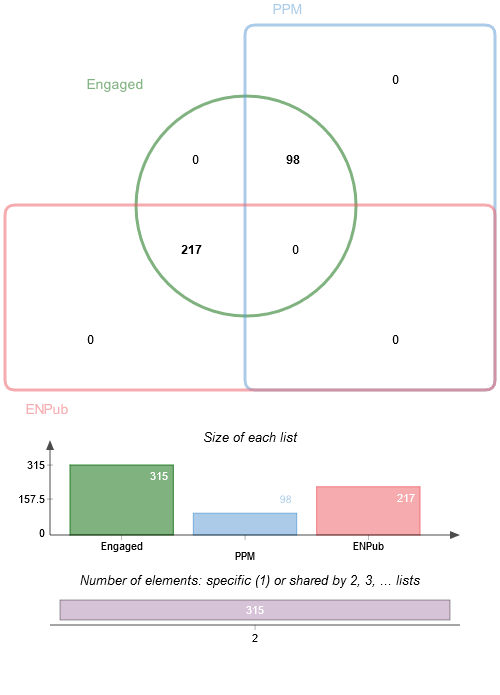


The LSTBLCP DOTS facilities list (red crosses) contained 309 HF. The Lagos TB drug procurement system (Pick’n’Pack) listed 298 facilities as engaged DOTS facilities (black dots) (See Figure 2). There were 274 HF that were common to both DOTS lists and an additional 59 HF that appeared on only one official DOTS centre list.

Figure 2: Comparison of STBLP list of Engaged Facilities and Pick’n’Pack list of engaged facilities.


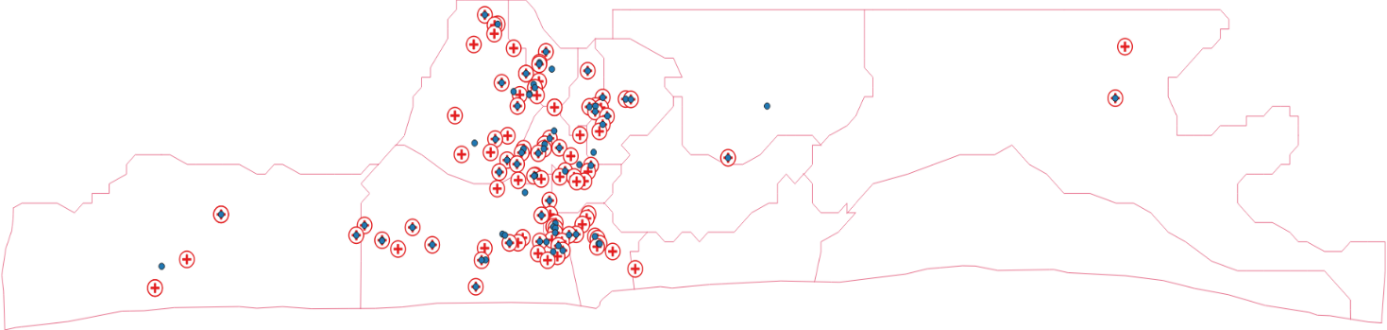


Figure 2: Map of the Lagos Private sector health facilities engaged TB service delivery as PPM or “engaged” facilities


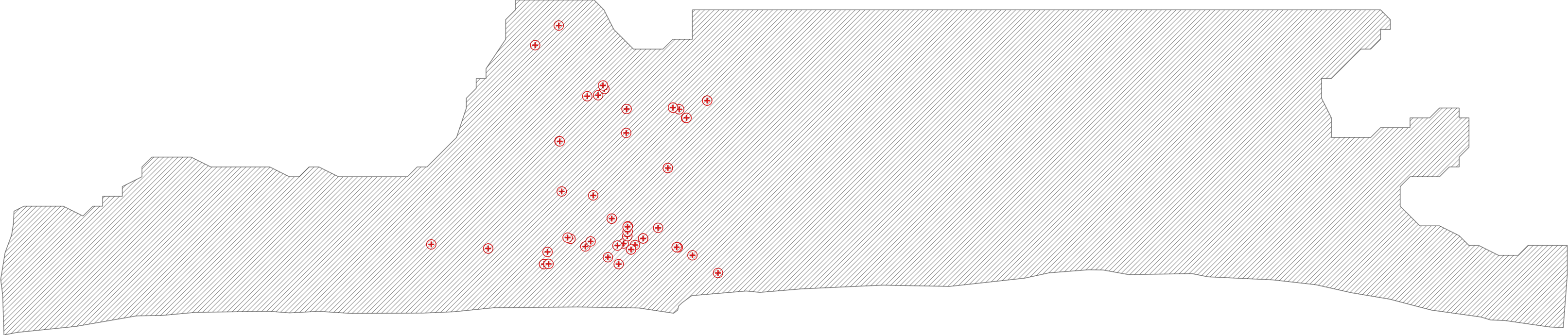


Creating an accurate sampling frame of Lagos private sector HF was challenging, but fortunately, multiple censes had recently been undertaken and could be leveraged. The unengaged private provider sampling frame was derived from three databases of HF in Lagos State: Health Facility Monitoring and Accreditation Agency (HEFAMAA), Millennium Development Goals (MDG) and Strengthening Health Outcomes through the Private Sector (SHOPS).[11] De-duplification yielded 2,634 unengaged private HF. A slight majority (56.2%) were classified as primary level (n=1,480), 40.2% were categorized as secondary level HF (n=1,058), 3.7% (n=96) had conflicting level classifications in different data sets and we thus labeled as unknown.

Table 1: Unengaged private health facilities in Lagos by facility level

| Primary level HF n (%) | Secondary level HF n (%) | unknown level HF n (%) | Total unengaged HF  n (%) |
| --- | --- | --- | --- |
| 1,480 (56.2) | 1,058 (40.2) | 96 (3.7) | 2,634 (100) |

The MDG sampling frame (below) is illustrative of the challenges of sampling unengaged HF. Approximately 36% of secondary level facilities and 32% of primary level facilities allege provision of TB treatment.

Table 2: MDG Survey: Private unengaged providers self-reported capacity to offer TB treatment (2012)

|  | HF without TB treatment | | HF reportedly with TB treatment | |  |
| --- | --- | --- | --- | --- | --- |
|  | n | % | n | % | TOTAL |
| Health Post Dispensary | 7 | 46.7% | 8 | 53.3% | 15 |
| Primary Health Clinic | 74 | 70.5% | 31 | 29.5% | 105 |
| Primary Healthcare Centre | 59 | 68.6% | 27 | 31.4% | 86 |
| Ward Model PHC Centre | 5 | 71.4% | 2 | 28.6% | 7 |
| Comprehensive Health Centre | 46 | 62.2% | 28 | 37.8% | 74 |
| Cottage Hospital | 36 | 69.2% | 16 | 30.8% | 52 |
| General Hospital | 10 | 66.7% | 5 | 33.3% | 15 |
| Maternity | 71 | 75.5% | 23 | 24.5% | 94 |
| Specialist Hospital | 80 | 55.9% | 63 | 44.1% | 143 |
| Teaching Hospital | 0 | 0.0% | 1 | 100.0% | 1 |
| TOTAL HF | 388 | 65.7% | 204 | 34.3% | 592 |

Stratified sampling of private unengaged HF by level was undertaken although it was not always possible to reliably stratify the sites by level due to discrepant strata classifications. The absence of strict naming conventions implied that HFs could label themselves “hospital” even in the absence of the commonly accepted features of a hospital (e.g. inpatient treatment). Nevertheless, oversampling secondary level facilities was prudent as this stratum had higher likelihood of TB provision and misreporting relative to other strata, so even small errors could have a large impact on estimates of the overall magnitude of under-notified TB treatment. While missing TB notifications was unlikely, undercounting treated TB cases in the unengaged private sector was the major validity threat to the study. Therefore we sampled 17.6% secondary level HF versus 9.5% of primary level HF. In total we sought to recruit 11% of all unengaged HF, 57% from secondary level HF and 43% from primary level HF.

Table 3: Sampling quotients for unengaged private facility strata

|  | Primary level health facilities  N=1,480 | Secondary level health facilities  N=1,059 (%) | Total  N=2634 (%) |
| --- | --- | --- | --- |
| Self-reported TB service provision[18] | 32% | 36% | 34.3% |
| Estimation of mis-reporting | 93% | 90% |  |
| Precision | 0.10 | 0.10 | 0.10 |
| Sampling Quotient | 9.5% | 17.6% | 11% |
| Raw sample size | 94 | 123 | 217 |
| Design effect | 1.5 | 1.5 | 1.5 |
| Sample size adjusted for design effect | 141 | 186 | 327 |

*The 95 HF with unconfirmed or discrepant facility level were not included in the initial sample, but some were included as part of the replacement sample pool, chosen to ensure geographical balance.

Table 4: Comparison of unengaged private HF sample and frame by level

|  | Primary level HF | | Secondary level HF | | Unconfirmed/  discrepant level | | Total | |
| --- | --- | --- | --- | --- | --- | --- | --- | --- |
|  | n | % | n | % | n | % | N | % |
| Universe of unengaged private HF | 1,480 | 56% | 1,058 | 40% | 96 | 4% | 2,634 | 100% |
| Sample size selected | 141 | 43% | 186 | 57% | 0 | 0% | 327 | 100% |

## Comparison of per protocol sampling plan and actual HF recruitment by facility type

A total of 608 facilities (86%) were recruited into the study out of which 564 (92.7%) granted full access to facility records and 44 (7.3%) granted partial access to facility records. Recruitment followed the per protocol sampling plan in terms of facility type and facility level, but geographical distribution varied in laboratories due to high frequency of substitution due to ineligibility (See Table 37).

Recruitment among the ambiguously unengaged public facilities was modest, with six of 23 HFs not participating (26.1%), primarily due to initial misclassification.

We anticipated substantial misclassification of facility level, facility functionality, and study eligibility among 2,634 unengaged private facilities because naming of facilities is unregulated, and the sector is highly dynamic. Eligibility was verified before fieldwork and again during field work. Nevertheless, HF closure was not uncommon (11.8%). HF closures and refusals were more frequent among HF that had contradictory level classifications on health facility lists. Among well characterized HF, the participation rate was 92.5% among primary level HF and 88.0% among secondary level HF. Refusal among the unengaged private HF was moderate (9.4%) overall and varied little by level (6.3% primary 9.9% secondary). Reasons for refusal were not captured routinely. Refusals were replaced with unengaged private HF of similar level and LGA where possible. The 77 HF with unconfirmed or discrepant facility level were not formally included in the sampling strategy, but 56 HF were approached during the course of sampling with replacement by LGA.

#### Table 14: Per protocol versus actual unengaged private sector inclusion by reason for non-participation

|  | **Primary**  **level HF** | | | **Secondary**  **level HF** | | **Unconfirmed/**  **contradictory level** | | **Total** | |
| --- | --- | --- | --- | --- | --- | --- | --- | --- | --- |
| **Total HF approached** | 142 | 100.0% | 182 | | 100.0% | 56 | 100% | 380 | 100% |
| **HF included** | 124 | 87.3% | 155 | | 85.2% | 20 | 35.7% | 299 | 78.7% |
| **HF refusal** | 9 | 6.3% | 18 | | 9.9% | 9 | 16.1% | 36 | 9.5% |
| **HF ceased operation** | 9 | 6.3% | 9 | | 4.9% | 27 | 48.2% | 45 | 11.8% |

Figure 28: Unengaged private HF recruitment by level

we summarize the way that the actual sample was generated, being careful to distinguish between refusal and ineligibility.

Recruitment followed the sampling plan in terms of facility type, facility level, and geographical distribution.

#### Table 5: Total HF participation by engagement status and reason for non-participation

| **Engaged DOTS** | **Participation**  **n (%)** | **Refusal**  **n (%)** | **Ceased Operation**  **n (%)** | **Total**  **n** |
| --- | --- | --- | --- | --- |
| Public engaged | 206 (98.2) | 4 (1.9) | 0 (0.0) | 210 |
| Private engaged | 85 (90.0) | 6 (6.4) | 3 (3.2) | 94 |
| Public unengaged | 17 (74.0) | 6 (26.0) | 0 (0.0) | 23 |
| Private unengaged | 299 (78.7) | 36 (9.5) | 45 (11.8) | 380 |
| **Total** | **608** | **16** | **82** | **706** |

#### Table 6: Total HF Participation by facility level and reason for non-participation

| **Facility level** | **Participation**  **n (%)** | **Refusal**  **n (%)** | **Ceased Operation n (%)** | **Total**  **n (%)** |
| --- | --- | --- | --- | --- |
| Primary | 362 (95.3) | 10 (2.6) | 8 (2.1) | 380 (53.8) |
| Secondary | 29 (96.7) | 1 (3.3) | 0 (0.0) | 30 (4.2) |
| Tertiary | 5 (100.0) | 0 (0.0) | 0 (0.0) | 5 (0.7) |
| Unconfirmed/contradictory | 212 (72.9) | 5 (1.7) | 74 (25.4) | 291 (41.2) |
| **Total** | **608** | **16** | **82** | **706** |

#### Table 7: Total HF Participation by ownership

| **Ownership** | **Participation**  **n (%)** | **Refusal**  **n (%)** | **Ceased Operation**  **n (%)** | **Total**  **n (%)** |
| --- | --- | --- | --- | --- |
| Public | 223 (96.1) | 9 (3.9) | 0 (0.0) | 232 (32.9) |
| Private | 385 (81.2) | 7 (1.5) | 82 (17.3) | 474 (67.1) |
| **Total** | **608** | **16** | **82** | **706** |

#### Table 8: Comparison of Per Protocol Sampling Plan and Actual Recruitment by facility type

| **Variable** | **Health Facilities** | | **Laboratories** | |
| --- | --- | --- | --- | --- |
|  | **Projected for inclusion**  **n (%)** | **Participating**  **n (%)** | **Projected for inclusion**  **n (%)** | **Participating**  **n (%)** |
| **Type of facilities** | | | | |
| Public | 217 (34.7) | 206 (33.9) | 43 (12.3) | 40 (12.2) |
| Private | 395 (65.3) | 402 (66.1) | 306 (87.7) | 288 (87.8) |
| Total | 605 (100.0) | 608 (100.0) | 349 (100.0) | 328 (100.0) |
| **Facility Level** | | | | |
| Primary | 176 (29.1) | 173 (28.5) | 18 (5.2) | 17 (5.2) |
| Secondary | 29 (4.8) | 28 (4.6) | 20 (5.7) | 19 (5.8) |
| Tertiary | 5 (0.8) | 5 (0.8) | 5 (1.4) | 4 (1.2) |
| Private | 395 (65.3) | 402 (66.1) | 306 (87.7) | 288 (87.8) |
| **Total** | 605 (100.0) | 608 (100.0) | 349 (100.0) | 328 (100.0) |

#### Table 9: Comparison of Per Protocol Sampling Plan and Actual Recruitment by LGA

| **LGA** | **sampling plan**  **n (%)** | **Study Participation**  **n (%)** | **Refusal**  **n (%)** | **Ceased Operation**  **n (%)** | **Total**  **n** |
| --- | --- | --- | --- | --- | --- |
| Agege | 27 (4.5) | 27 (90.0) | 0 (0.0) | 3 (10.0) | 30 |
| Ajeromi/Ifelodun | 39 (6.4) | 38 (86.4) | 1 (2.3) | 5 (11.4) | 44 |
| Alimosho | 81 (13.4) | 89 (96.7) | 1 (1.1) | 2 (2.2) | 92 |
| Amuwo odofin | 32 (5.3) | 26 (83.9) | 2 (6.5) | 3 (9.7) | 31 |
| Apapa | 16 (2.6) | 14 (63.6) | 1 (4.5) | 7 (31.8) | 22 |
| Badagry | 12 (2.0) | 12 (100.0) | 0 (0.0) | 0 (0.0) | 12 |
| Epe | 10 (1.7) | 11 (84.6) | 2 (15.4) | 0 (0.0) | 13 |
| Eti osa | 19 (3.1) | 17(89.5) | 0 (0.0) | 2 (10.5) | 19 |
| Ibeju lekki | 6 (1.0) | 7 (77.8) | 0 (0.0) | 2 (22.2) | 9 |
| Ifako Ijaye | 24 (4.0) | 26 (92.8) | 1 (3.6) | 1 (3.6) | 28 |
| Ikeja | 28 (4.6) | 31 (94.0) | 1 (3.0) | 1 (3.0) | 33 |
| Ikorodu | 23 (3.8) | 23 (92.0) | 0 (0.0) | 2 (8.0) | 25 |
| Kosofe | 21 (3.5) | 54 (83.1) | 0 (0.0) | 11 (16.9) | 65 |
| Lagos Island | 20 (3.3) | 20 (83.3) | 0 (0.0) | 4 (16.7) | 24 |
| Lagos Mainland | 27 (4.5) | 19 (67.9) | 1 (3.6) | 8 (28.6) | 28 |
| Mushin | 34 (5.6) | 27 (65.9) | 0 (0.0) | 14 (34.1) | 41 |
| Ojo | 50 (8.3) | 40 (88.9) | 3 (6.7) | 2 (4.4) | 45 |
| Oshodi/Isolo | 24 (4.0) | 45 (95.7) | 2 (4.3) | 0 (0.0) | 47 |
| Shomolu | 55 (9.1) | 24 (85.7) | 0 (0.0) | 4 (14.3) | 28 |
| Surulere | 27 (4.5) | 58 (82.9) | 1 (1.4) | 11 (15.7) | 70 |
| **Total** | **605 (100)** | **608 (86.1)** | **16 (2.3)** | **82 (11.6)** | **706** |

#### Table 10: Participation by laboratories according to local government area (LGA)

| **LGA** | **Stand-alone and embedded laboratories** | |
| --- | --- | --- |
|  |  |  |
|  | **Projected for inclusion** | **Participating** |
|  | n (%) | n (%) |
| Agege | 18 (5.2) | 14 (4.3) |
| Ajeromi | 19 (5.4) | 13 (4.0) |
| Alimosho | 54 (15.5) | 43 (13.1) |
| Amuwo Odofin | 20 (5.7) | 15 (4.6) |
| Apapa | 5 (1.4) | 4 (1.2) |
| Badagry | 8 (2.3) | 5 (1.5) |
| Epe | 3 (0.9) | 3 (0.9) |
| Eti Osa | 14 (4.0) | 8 (2.4) |
| Ibeju Lekki | 1 (0.3) | 1 (0.3) |
| Ifako Ijaye | 10 (2.9) | 6 (1.8) |
| Ikeja | 23 (6.6) | 26 (7.9) |
| Ikorodu | 17 (4.9) | 17 (5.2) |
| Kosofe | 20 (5.7) | 18 (5.5) |
| Lagos Island | 9 (2.6) | 8 (2.4) |
| Lagos Mainland | 17 (4.9) | 12 (3.7) |
| Mushin | 12 (3.4) | 11 (3.4) |
| Ojo | 33 (9.5) | 53 (16.2) |
| Oshodi isolo | 16 (4.6) | 27 (8.2) |
| Shomolu | 13 (3.7) | 15 (4.6) |
| Surulere | 37 (10.6) | 29 (8.8) |
| **Total** | **349 (100)** | **328 (100)** |

WHO recommends data be collected for a three-month sample window for prospective inventory studies when there are no electronic case-based records. However, anecdotal reports and descriptive analyses suggested seasonality and high variability in notifications throughout the year, and an average variation of 54% between quarters. Some program coordinators appear to use the last quarter of the year as a “mop-up” quarter to notify cases that were missed earlier. Others appear to use the first quarter of the year for this purpose. High variability would make the selection of a single quarter subjective and could introduce bias into the estimates of under notification. Therefore, we chose to collect data for 12 months, with a three month buffer.

Figure 4: seasonal variation in TB notifications in Lagos State 2015
