# Supplementary material for: Hybrid Approach to Estimation of Underreporting of Tuberculosis Case Notification in High-Burden Settings With Weak Surveillance Infrastructure: Design and Implementation of an Inventory Study
Source: JMIR Public Health Surveill. 2021 Mar 15;7(3):e22352. doi: 10.2196/22352 (PMC8088841; doi:10.2196/22352)
Supplement: Multimedia Appendix 3 [file publichealth_v7i3e22352_app3.pdf]

Lagos Inventory Study			
TITLE: Standard Operating Procedures PILOT			
**#006**	rev. 00	Effective Date: 11/06/2017	Supersedes rev. NA

Submitted by:	Reviewed by:	Approved by:
(Signature & Date)<br>Name: Adejumo Olusola<br>Title: Principal Investigator	(Signature & Date)<br>Name:<br>Title:	(Signature & Date)<br>Name: Ellen M.H. Mitchell<br>Title: Co-Principal Investigator

PURPOSE (or OBJECTIVE)

The purpose of a pilot is to identify weaknesses and to strengthen them for the actual study. The eight specific objectives of the pilot are to:

1) Identify **challenges and solutions** related to community entry, informed consent, and access (including transport, refusals, delayed admissions, rate of re-visitation)
2) Estimate the **length of time and effort** required to complete specific tasks
3) Assess **functionality** of the web-based data application in the field (e.g. stability of internet access, find and fix bugs,
4) Pilot test and adapt the draft **case verification process** using field data in engaged facilities
5) Pilot test **all the questionnaires** for length, acceptability (AKA respondent burden), intelligibility, feasibility
6) Measure the **competence** of the Data officers in finding TB patients that were not notified and (where required) identify areas for refresher training and reinforcement
7) **Compare** the TB cases collected with the TB cases notified to detect under-notification and over-notification
8) Assess what type of support (incentives, training, provision of forms and registers) might be useful at these sites to improve case notification?

SCOPE

This SOP applies to all study team members participating in research activities for the Lagos Inventory study.

RESPONSIBILITIES

Data Officers, Data Managers, and Investigators are responsible for following this SOP and ensuring the respectful, ethical, and responsive interactions with stakeholders at all levels of the health facility. Oversight of this Standard Operating Procedure (SOP) ensuring that informed consent and facility entry steps are followed at all times: Principal Investigator (PI). Maintaining confidentiality and adhering to this SOP: Data manager and all data officers

MATERIALS REQUIRED

1. a letter of introduction from the Lagos State Ministry of Health.
2. Copies of the ethical approval letters from the LSTBLCP and LASUTH
3. data collector identification badge?
4. informed consent forms
5. field notebooks for data collectors
6. Tables - charged

NB: The letters of introduction to the sites will be personalized (i.e. with the name and address of the site)and that they be on quality paper and a real signature.. Each letter will be in an envelope also bearing the name and address.

PROCEDURES

The pilot study will implement the approved SOPs for the purpose of detecting their shortcomings and determining how they can be improved. Data collectors are required to keep a field notebook with notes.

Data Officers will go to 2 facilities per type to implement the SOPS, as follows.

	Levels	Number of pilots sites
Public engaged	hospitals	2
	clinics	2
Private engaged	hospitals	2
	clinics	2
Private unengaged	hospital	2
	clinics	2
Engaged laboratories		2
Unengaged stand alone laboratories		2
	**FACILITIES**	**16**

At each site, they will work on all 8 objectives. In addition to filling out the regular forms, they will also fill the field notes form (see appendix).

1) **Objective 1:** Identify **challenges and solutions** related to community entry, informed consent, and access
    a. How accurate/useful are the GIS maps for reaching the facilities?
    b. How hard is it to navigate the hospital or facility records
2) **Objective 2:** Estimate the **length of time and effort** required to complete specific tasks(including transport, refusals, delayed admissions, rate of re-visitation)
    a. Doe the length of time spent per site per type of facility match our estimates?
3) **Objective 3:** Assess **functionality** of the tablets, the internet, and the web-based data application in the field (e.g. stability of internet access, find and fix bugs,)
    a. We will document how *often and under what conditions the internet fails,*
    b. We will employ the back-up paper forms, and these forms will also be tested for *utility, time-burden.*
4) **Objective 4:** Pilot test and adapt the draft **case verification process** using field data in engaged facilities
    a. How difficult is compliance with the matching protocol?
    b. What are the main reasons for over-reporting?
    c. What are the main reasons for under-reporting?
5) **Objective 5:** Pilot test **all the questionnaires** for length, acceptability (aka respondent burden), intelligibility, feasibility
    a. What is the min, max, and range of the time required for each type of questionnaire
6) **Objective 6:** Measure the **competence** of the Data officers in finding TB patients that were not notified and (where required) identify areas for refresher and reinforcement
7) **Objective 7: Compare** the cases collected with the cases notified to detect under-notification and over-notification
8) **Objective 8:** Assess what type of support (incentives, training, provision of forms and registers) might be useful at these sites to improve case notification?

FIELD VISIT NOTES

Facility name:
Data collector Name:
Date:

What challenges did you experience?

	Challenges	solutions
Facility entry and consent		
Identifying TB patients in the facility		
Recording data about the TB patients in web based application		
Use of paper forms		
Conducting Interviews with staff		

How often and under what conditions did the internet fail?,
How long did it take to get to the site?

How long did you spend at the site?
How much of this time was spent waiting to begin?
What took the most time?

<table>
<tr><th colspan="4">Lagos Inventory Study</th></tr>
<tr><td colspan="4">TITLE: Standard Operating Procedures for data collection in engaged health facilities</td></tr>
<tr><td>#001</td><td>rev. 00</td><td>Effective Date: 19/05/2017</td><td>Supersedes rev. NA</td></tr>
<tr><td colspan="4">Page 1 of x</td></tr>
<tr><td>Submitted by:
_____
(Signature & Date)
Name:
Title: Principal Investigator</td><td colspan="2">Reviewed by:
_____
(Signature & Date)
Name:
Title:</td><td>Approved by:
(Signature & Date)
Name: Ellen M.H. Mitchell
Title: Senior Epidemiologist</td></tr>
</table>

PURPOSE (or OBJECTIVE)

The purpose of this SOP is to define what data collectors must do to:

- verify notified TB cases captured in the web based application using the engaged health facility TB register
- enter TB patients treated in 2015 by the facility *who are not yet notified*

SCOPE

This SOP applies to all data Officers (DO) responsible for identifying TB patients treated in the engaged DOTS facilities in the Lagos inventory study.

TERMS AND DEFINITIONS

Informed consent:	Is a process for getting permission before conducting an intervention on a person. An informed consent can be said to have been given based upon a clear appreciation and understanding of the facts, implications, and consequences of an action
Definitive match:	A deterministic match of the seven key variables- (i.e. 100% agreement of all 7, exact spelling match).
Fuzzy match:	A probabilistic match of the seven key variables (i.e. agreement of all 7, but with only minor spelling variations in the name fields).
A non-match	Any variation in the 7 key variables- beyond spelling.
Verification:	The process of comparing two sets of variables about a TB patients to determine if they may represent the same person.

MATERIALS REQUIRED

Data verification at engaged DOTS facilities will be done using:

7. a letter of introduction from the Lagos state Ministry of Health
8. Copies of the ethical approval letters from the LSTBLCP and LASUTH
9. Letter of permission from Chief Medical Directors to collect data from DOTS facility and other department in the hospital.
10. data collector identification
11. copies of revised TB registers
12. tablets or laptop with camera is at least 80% charged
13. Internet access
14. paper based format (for use in the event of internet problem)

RESPONSIBILITIES

a) Photocopy facility registers – PI
b) Obtain permission from the Chief Medical Director of each public secondary and tertiary hospitals to collect data from the DOTS facility and other department in the hospitals - PI

PROCEDURES

The following parts will be taken by data Officers during verification of TB log books at DOTS facilities.

PART 1: COMMUNITY ENTRY & INFORMED CONSENT

Refer to the SOP for Community entry and informed consent

1. Data Officer will introduce themselves and provide the provider the letter of introduction and ethical approval from the LSTBLCP and LASUTH respectively.
2. Data Officer must also provide the DOTS provider letter of permission from the Chief Medical Director to obtain from the DOTS facility
3. Data Officer must take informed consent before data verification and data collection in each health facility (See SOP for Informed consent)
4. If the DOTS provider refused Data Officers from collecting data in the DOTS facility, the Data Officer must inform the Data Manager immediately via telephone.
5. If the Data Officer cannot get the data manager on the phone, he/she must call the PI.
6. If both of them cannot be reached on the phone, the Data Officer must send a mail to the Data Officer and copy the PI.
7. In the event that Data Officers are asked to return for data collection, they must ask of the date, time convenient for the DOTS provider, name of DOTS provider and phone number before leaving.
8. The Data Officer must inform the Data Manager via mail, sms or phone call of the rescheduled visit, reason for the revisit. name of the facility, name of the and phone number of DOTS provider.

PART 2: FACILITY SUMMARY REPORT

Data Officers will ensure that tablet is at least 80% charged and there is good internet access before data capture. If no internet is available, the FACILITY SUMMARY REPORT will be done on paper.

The DO will first complete a "facility summary report" describing the facility and verifying the data from the sampling frame.

1.1. LGA of facility,
1.2. name of facility,
1.3. Verify that it is *private* (y/n)and *unengaged(y/n)* and *operational in 2015(y/n).*
1.4. Record the departments present within the facility. For example:
    i) Medical*(y/n).*,
    ii) Paediatrics*(y/n).*,
    iii) Surgical, in patient*(y/n).*
    iv) Obstetrics and Gynaecology*(y/n).*,
    v) Morgue*(y/n).*
    vi) HIV Clinic*(y/n).*
1.5. Indicate in how many of the existing departments data access was granted and patient records were searched.
1.6. An aggregate count of how many TB patients were found in each department.
1.7. Indicate the primary data source(S) consulted (E.g. case notes, registers, files)

PART 3:VERIFICATION OF NOTIFIED TB CASES AT ENGAGED DOTS FACILITY

- Data Officer will attempt to match specific patient variables entered from the central registers using the facility register.
- Definitive Match will ensure all the following 7 variables match exactly.
    1. First name,
    2. Surname,
    3. Age,
    4. Gender,

5. Date treatment started,
6. Smear status
7. Treatment outcome

- Minor spelling variations in names of the patient will be considered as “probable” matches. Minor spelling variations include:
    - Inverting the order of first and last names
    - Abbreviations of common names (-e.g. Olusola and Sola will be considered the same name.)
    - British Commonwealth orthography or American orthography
    - Slight differences in word division- the presence or absence of spaces between words in a name.
    - The absence of letters that are not pronounced in names.
    - Slight spelling differences names (such as dropping double consonants)
- All other types of discrepancies will prompt the entry of a new record using web based application
- In the event of poor or lack of internet access, data Officers shall collect all patient’s variables on a paper based format and verification can be done when good internet access is established.
- Data obtained with paper based format must be entered in the web based application whenever there is good internet access not exceeding 24 hours after initial data collection.
- If the 2015 TB health facility register could not be found at the health facility. The Data Officer shall indicate on the web based application that verification could not be done because of missing facility register and inform the Data Manager.
- The Data Manager on receiving such information of missing facility register shall inform the PI who will call the DOTS provider to confirm and also notify the State TB program officer of the incident.

A. Data collection in other department in Engaged Health facilities

NB: All the procedure for community entry in PART 1 above also apply here

- Within each department,
    a) Whether the key variables found in the department patient records match those of the central TB register – deterministically or probabilistically or not using the criteria set in **A** above
    b) For TB Patients treated in 2015 at the facility who were ***not found*** in the central TB registers but rather in other sites, 9ine key variables of each TB patient will be recorded
        1. First name,
        2. Surname,
        3. Age,
        4. Gender,
        5. Date treatment started
        6. Smear status (or Gene Xpert status)
        7. Treatment outcome
        8. If referred – where was he/she referred
        9. Primary source document (case notes, registers, discharge slip, DSNO)

1. An aggregate count of how many TB patients were found in each department.
2. Indicate the primary data source(S) consulted (e.g. case notes, registers, files)

- Data Officers will ensure that data obtained with paper based format must be entered in the web based application whenever there is good internet access not exceeding 24 hours after initial data collection.
- Data Officer will give the paper based format used to abstract data from other departments in engaged DOTS facilities to the data manager after entering the data using web application

APPENDIX A: TB register (2015 version)

APPENDIX B: **Proforma for data collection in Departments in Engaged Health facilities**

Facility name………………………………………Department: ____________ LGA………………………
Name of Data Officer: __________________

Nos	First name	Surname	Age (in years)	Gender M=1 F=2 n/a=77	Date treatment started	Smear or GeneXpert status	Treatment outcome	If referred - where
1								
2								
3								
4								
5								
6								
7								
8								
9								
10								

Lagos Inventory Study

TITLE: **Standard Operating Procedures for data collection in Unengaged private health facilities**

#002	rev. 00	Effective Date:19/05/2017	Supersedes rev. NA

Page 1 of 4

Submitted by:	Reviewed by:	Approved by:
_____ (Signature & Date)<br>Name: Olusola Adejumo<br>Title: Principal Investigator	_____ (Signature & Date)<br>Name:<br>Title:	(Signature & Date)<br>Name: Ellen M.H. Mitchelll<br>Title: Senior Epidemiologist

PURPOSE (or OBJECTIVE)

The purpose of this SOP is to define what data collectors must do to enter TB cases in unengaged private health facility and training of the on R-R tool for TB reporting

SCOPE

This SOP applies to all data officers (DO) responsible for entering LGA TB registers in the Lagos inventory study and the Data Manager

RESPONSIBILITIES

a. Send sms to facility to explain the purpose and procedures of the study and solicit for verbal consent (PI)
b. Inform health facility of the likely date for visit (PI)

TERMS AND DEFINITIONS

Informed consent:	Is a process for getting permission before conducting an intervention on a person. An informed consent can be said to have been given based upon a clear appreciation and understanding of the facts, implications, and consequences of an action
Definitive match:	A deterministic match of the seven key variables- (i.e. 100% agreement of all 7, exact spelling match).
Fuzzy match:	A probabilistic match of the seven key variables (i.e. agreement of all 7, but with only minor spelling variations in the name fields).
A non-match	Any variation in the 7 key variables- beyond spelling.
Verification:	The process of comparing two sets of 7 variables about a TB patients.
Unengaged	Facilities without a current, formal affiliation with the national TB program
Minor spelling variations	Minor spelling variations include:<br>o Inverting the order of first and last names<br>o Abbreviations of common names (e.g. Olusola and Sola will be considered the same name.)<br>o British Commonwealth orthography or American orthography<br>o Slight differences in word division- the presence or absence of spaces between words in a name.<br>o The absence of letters that are not pronounced in names.

EQUIPMENT REQUIRED

1. the tablets or laptop is at least 80% charged

MATERIALS REQUIRED

1. a personalized letter of introduction from the Lagos State Ministry of Health
2. Copies of the ethical approval letters from the LSTBLCP and LASUTH
3. Letter from chairman of the Association of General and Private Practitioners (HEFAMMA)
4. data collector personal identification
5. tablet computer with camera-
    a. charged at least 80%
    b. with a clean memory card
6. blank data collection forms and questionnaires for us in the event of internet outage.
7. DSNO notification forms
8. mini- version of revised TB treatment
9. mini-version of presumptive TB register
10. mini-version of TB lab registers
11. the phone number of the DSNO officer responsible for data collection from their facility

PROCEDURES

PART 1: COMMUNITY ENTRY & INFORMED CONSENT

Refer to the SOP for Community entry and informed consent

1. Data officers will introduce themselves and hand over the letter of introduction from LSTBLCP and HEFFEMA and ethical approval from LASUTH.
2. Data officer inform health facility representative of the purpose for visit and ask if TB patients were ever diagnosed or treated at the facility.
3. If the private provider informs the data officer that TB patients were ever treated at the facility in 2015, the data officer then proceeds to take informed consent. Data officer must take informed consent before data collection in private health facility (Please refer to SOP for informed consent)
4. If the private health provider refuses Data Officers from collecting data, the DO should ask why. It is valuable to know the reason for the refusal.

5. If TB has ever been diagnosed or treated, then they are asked to please consult their patient files to see if any TB patient was treated in 2015. A thorough check of the patient records can take time. The DO can offer to assist the staff, if that is agreeable to them. The DO should explain the definition of "TB patient treated."
6. If the representative answers "NO" to the question about whether or not TB patient was cared for in 2015 after checking records and case notes from 2015,
7. DO shall thank the private provider for their time and for checking their records. Data officer must inform the Study Coordinator immediately via telephone in the event that the private provider requires more information to make an informed decision on consent..
8. DO shall offer to share resources with the private provider of on how to report TB to the DSNO officer or the LSTBLCP and train the representative on R-R tool **(see SOP on training on R-R tool)**
9. In the event that Data Officers were asked to return for data collection, they must ask of the date and time that is convenient for the private health provider before leaving.
10. The Data officer must inform the Data Manager via mail, sms or phone call of the rescheduled visit, reason for the revisit and name of the facility,

PART 2: FACILITY SUMMARY REPORT

The DO will first complete a "facility summary report" describing the facility and verifying the data from the sampling frame.

1.8. LGA of facility,
1.9. name of facility,
1.10. Verify that it is *private* (y/n)and *unengaged(y/n)* and *operational in 2015(y/n).*
1.11. Record the departments present within the facility. For example:
    vii) Medical*(y/n).*,
    viii) Paediatrics*(y/n).*,
    ix) Surgical, in patient*(y/n).*
    x) Obstetrics and Gynaecology*(y/n).*,
    xi) Morgue*(y/n).*
    xii) HIV Clinic*(y/n).*
1.12. Indicate in how many of the existing departments data access was granted and patient records were searched.
1.13. Indicate the primary data source(S) consulted (e.g. case notes, registers, files)

PART 3: DATA CAPTURE OF TB PATIENTS MANAGED AT UNENGAGED PRIVATE HEALTH FACILITIES

Tablets with web based application will be used to abstract TB patient information from patient notes and records at

11. Data entry officer will indicate *the primary data source* and whether access was to all primary data sources was granted
12. Data collectors will abstract the following <u>14 variables</u> from facility records:
    1) Surname,
    2) first name,
    3) age,
    4) gender,
    5) LGA of patient residence
    6) smear result,
    7) gene Xpert result,
    8) Chest x-ray conducted (y/n)
    9) Chest x-ray result (TB/no TB)
    10) date of commencement of TB treatment,
    11) treatment outcome
    12) Facility referred for treatment, if referred.
    13) Department where the case was found
    14) Primary source document (case notes, registers, discharge slip, DSNO)

using the web based application or the paper based forms.

13. Data officers will not bother private providers on missing variables, except if the variables on diagnosis (smear, chest x-ray) are absent Names and diagnostic variables are essential.
14. In the event of poor or lack of internet access, data officers shall collect all patient's variables on a paper based format and subsequently entered on the web based application when good internet access is established.

PART 3: FACILITY INTERVIEW

1. DO will conduct the interview in a place with audio and visual privacy
2. Data Officer shall read the instructions aloud, and re-explain confidentiality provisions before starting the interview
3. Even though we do it on the tablet, there will be times when we have to do it on paper.
4. the questionnaire for private health provider after taking informed consent.

PART 4: TRAINING ON RECORDING AND -REPORTING TOOLS

1. The data officer shall give;
   a. DSNO notification forms
   b. mini- version of revised TB treatment
   c. mini-version of presumptive TB register
   d. mini-version of TB lab registers

   to the representative of the private health facility.

1. The data officer will explain all the variables on the forms to the representative of private health facility and also explain the pathways of notification.
2. The data officer will provide the private health provider with the phone number of the DSNO officer responsible for data collection from their facility.

PART 4: TRANSMISSION OF DATA TO CENTRAL STUDY SERVER

The following PARTs should be taken to ensure data capture is of high quality

1. Data of TB patients found in unengaged private health facilites will be abstracted by the health facility representatives on a paper based format
2. The DO may assist in abstracting relevant details of TB patients found at the facility if the health facility representative permit them.
3. The health facility representative must sign to confirm the number of patients abstracted on the paper based format are those found at the health facility
4. The DO must enter the details of patients abstracted on the paper based format on the web based application within 24 hours of collection from the health facility representative
5. The DO must return the paper based format received from the health facility representative to the Data Manager within 24 hours
6. The data manager must ensure safe keeping of all paper based format received from the DO

NB: If the there is a laboratory in the health facility, data of smear positive TB patients ALONE will be collected from the laboratory register **(refer to SOP for Unengaged Laboratory)**

<table>
<tr><td colspan="4">Lagos Inventory Study</td></tr>
<tr><td colspan="4">TITLE: Standard Operating Procedures for data collection in engaged Laboratories</td></tr>
<tr><td>#003</td><td>rev. 00</td><td>Effective Date: 05:05:2017</td><td>Supersedes rev. NA</td></tr>
<tr><td colspan="4">Page 1 of x</td></tr>
</table>

Submitted by:	Reviewed by:	Approved by:
_____<br>(Signature & Date)<br>Name: Olusola Adejumo<br>Title: Principal Investigator	_____<br>(Signature & Date)<br>Name:<br>Title:	(Signature & Date)<br>Name: Ellen M.H.Mitchell<br>Title:

1. PURPOSE (or OBJECTIVE)

The purpose of this Standard Operative Procedure (SOP) is to provide the data officers with uniform approach to using web based application to enter data from laboratory registers of engaged DOTS facilities.

2. SCOPE

This SOP applies to all data officers (DO) responsible for entering Laboratory registers of engaged DOTS facilities in the Lagos inventory study. These procedures are to be followed after successful facility entry and informed consent procedures are completed.

3. RESPONSIBILITIES

a. Ensuring availability facility laboratory register to data officer – Principal investigator
b. Numbering of pages of laboratory register – Data Manager (DM)
c. Record keeping of total number of smear positive TB cases and Xpert positive in each laboratory register – DM
d. Ensuring tablets and laptops are functional – DM
e. Ensuring availability of good internet access – DM
f. Provision of personal id and password for data officers – DM
g. Develop plan for the number of laboratory register be entered by each data officer – DM
h. Safe keeping of copies of laboratory registers - DM

4. ABBREVIATIONS

PI: Principal investigator
SOP: Standard Operating Procedure
TB: Tuberculosis
DM: Data manager
LGA: Local Government Area
HQ: Headquarters

TERMS AND DEFINITIONS

Record	Any item, collection, or grouping of information that includes protected health information and is maintained, collected, used, or disseminated by healthcare professionals.
Web based application	A web-based application is any program that is accessed over a network connection using Hypertext transfer Protocol (HTTP), rather than existing within a device's memory. Web-based applications often run inside a web browser. However, web-based applications also may be client-based, where a small part of the program is downloaded to a user's desktop, but processing is done over the internet on an external server.

Laboratory Register	A book or records containing list of names of presumptive TB cases that did smear microscopy or gene Xpert
Laboratory sputum Central register	Records containing list of names and smear results TB presumptive cases and follow up smear results of TB patients on treatment in Engaged DOTS facilities
Gene Xpert Register	Records containing list of names and Xpert result of presumptive TB cases
DOTS	Directly Observed Treatment Short course
Functional laptop	Working laptop
Good internet connection	Internet connectivity is strong enough for the web based application (usually presence of 3G network)
Safe keeping	Register kept in the safety cabinet

MATERIALS REQUIRED

a. Laptops should be at least 80% charged
b. Tablets at least 80% charged
c. Copies of LGA TB Central registers
d. Internet access
e. paper based format

PROCEDURES

PART 1: DIFFERENTIATION OF *DIAGNOSTIC* SMEARS vs *FOLLOW-UP* SMEARS

1. Data officer shall check the column "Examination for" on the register to distinguish between diagnosis and follow up sputum.
2. Data officers shall consider the test as diagnostic if the column under 'D' is ticked.
3. In the event that the column for follow-up and diagnosis was left blank, data officers shall check the number of sputum specimen submitted for test
4. If there were two sputum specimens submitted, data officer shall consider it to be for diagnosis. (only one specimen is usually collected for follow-up test).

PART 2: DATA CAPTURE OF LABORATORIES REGISTER AT ENGAGED DOTS FACILITIES

5. Identify the laboratory register with TB tests in it.
   a. Data officers will extract the following information on smear positive TB or Gene Xpert positive cases at diagnosis (ONLY) in the laboratory registers of 2015 using web based application:
   b. LGA, Name of facility, department where data was obtained,
   c. quarter of year,
   d. laboratory serial number,
   e. date of lab test,
   f. Surname of patient,
   g. First name,
   h. Age,
   i. Gender,
   j. LGA of residence
   k. Referring health facility
   l. type of lab test (Smear or Gene Xpert)

6. Patients with least one positive smear result will be considered as smear positive by the data officer whether it was: scanty, 1+,2+, or 3+
7. Patients with positive Follow up sputum test results shall not be entered by Data officers
8. Available patient data in the laboratory register will be entered by Data officers

9. Data manager will keep record of missing information on the laboratory register for decision making
10. Copies of laboratory register shall be kept safely in the cabinet by the data manager

PART 3: COLLECTING INFORMATION ON GENEXPERT POSITIVE CASES

11. Data officer will extract similar information from gene Xpert register on TB with positive gene Xpert result.
12. In the event that a patient's details appear on both the sputum and gene Xpert register and, the data officer should enter it twice.
1. Genexpert is never used for follow-up, so all Xpert test should be considered as diagnostic and all positives recorded.

PART 4: Solving Technical issues

13. When the laptop or tablet develops a fault, the data officer will inform the data manager who will try to solve problem over the phone.
14. If the challenge still persists, data manager will send another tablet to the data officer on the field (if the distance is not far) or ask the data officer to abstract data from the laboratory register on a paper based format containing similar variables on the web based application.
15. In situation that the web application is down, the data officer shall inform the data manager who will inform the IT expert in HQ. Data officer will continue data collection on a paper based format

APPENDICES

APPENDIX A: TB laboratory register

<table>
<tr><td colspan="6" align="center">Lagos Inventory Study</td></tr>
<tr><td colspan="6">TITLE: Standard Operating Procedures For Data Collection In Unengaged Stand-alone Laboratories</td></tr>
<tr><td colspan="2">#004</td><td colspan="2">rev. 00</td><td>Effective Date: 19/05/2017</td><td>Supersedes rev. NA</td></tr>
<tr><td colspan="6" align="right">Page 1 of 3</td></tr>
<tr><td colspan="2">Submitted by:

_____
(Signature & Date)
Name: Olusola Adejumo
Title: Principal Investigator</td><td colspan="3">Reviewed by:

_____
(Signature & Date)
Name:
Title:</td><td>Approved by:

(Signature & Date)
Name: Ellen M.H. Mitchell
Title: Senior Epidemiologist</td></tr>
</table>

1. **PURPOSE (or OBJECTIVE)**

The purpose of this Standard Operative Procedure (SOP) is to provide the Data Officers with the uniform approach to using the web based application to enter data from registers of unengaged stand-alone laboratories.

2. **SCOPE**

This SOP applies to all Data Officers (DO) responsible for entering register of unengaged stand-alone laboratory in the Lagos inventory study.

3. **RESPONSIBILITIES**
   a. Ensuring availability proforma line listing smear positive TB cases diagnosed in unengaged stand-alone laboratories – Principal Investigator (PI)
   b. Ensuring tablets and laptops are functional – DM
   c. Ensuring availability of good internet access – DM
   d. Provision of personal id and password for Data Officers – DM
   e. Develop plan for the number of laboratory register be entered by each Data Officer – DM.
   f. Safe keeping of copies of proforma – DM

g. Call the facility to explain the purpose and procedures of the study and solicit verbal consent (DM)
h. Share the invitation letter, proforma, and consent form (DO)
i. Collect completed proforma from laboratories (DO)

4. ABBREVIATIONS

PI: Principal investigator
SOP: Standard Operating Procedure
TB: Tuberculosis
DM: Data manager
LGA: Local Government Area
HQ: Headquarters

TERMS AND DEFINITIONS

Record	Any item, collection, or grouping of information that includes protected health information and is maintained, collected, used, or disseminated by healthcare professionals.
Web based application	A web-based application is any program that is accessed over a network connection using Hypertext tranfer Protocol (HTTP), rather than existing within a device's memory. Web-based applications often run inside a web browser. However, web-based applications also may be client-based, where a small part of the program is downloaded to a user's desktop, but processing is done over the internet on an external server.
Register	A book or records containing list of names
Laboratory sputum Central register	Records containing list of names and smear results TB presumptive cases and follow up smear results of TB patients on treatment in Engaged DOTS facilities
Gene Xpert Register	Records containing list of names and Xpert result of presumptive TB cases
DOTS	Directly Observed Treatment Short course

5. MATERIALS REQUIRED

1. Completed Paper proforma
2. Consent form
3. Personally-addressed Letter of invitation
4. Laptops / Tablets about 80% charged
5. Internet access
6. Proforma
7. Mini-lab registers

PROCEDURES

PART 1: LABORATORY SUMMARY REPORT

Data Officer will confirm eligibility and information from the sampling frame:

1. LGA of facility,
2. Name of laboratory,
3. Confirm – unengaged (y/n)
4. Confirm – operational in 2015 (y/n)

PART 2 : DATA CAPTURE AT UNENGAGED STAND ALONE LABORATORIES

16. Data Officer will enter the following 9 variables on smear positive TB or Gene Xpert positive cases at diagnosis (ONLY) from the paper-based proforma containing the 2015 line listing of TB patients diagnosed in unengaged stand-alone laboratory using web based application: There are 9 essential variables:
    1. date of lab test,
    2. Surname of patient,

3. First name of patient,
4. Age,
5. Gender,
6. LGA of residence
7. type of lab test (Smear or Gene Xpert)
8. Smear positivity -
9. Referring Health facility

17. Patients with one positive smear result will be considered as "new diagnosis smear positive" by the Data Officer regardless of smear positivity (scanty, 1+,2+3+).
18. In the event that one or more of the 9 essential variables are missing, the DO will engage the laboratory to try to fill in the gaps in information.
19. If that is unsuccessful, the DM shall be called to ask how to proceed.
20. DM will trouble shoot in real time – and keep record of missing information on the laboratory register for future decision making
21. Copies of laboratory register shall be kept safely in the locked cabinet by the DM

PART 3: DIFFERENTIATION OF SPUTUM SMEAR TEST FOR DIAGNOSIS AND SMEAR FOR FOLLOW -UP

22. Data Officer shall check the column for type of laboratory test on the proforma to distinguish between sputum smear test and gene Xpert test. Only one specimen is usually collected for follow up test, so if the person has only 1 sample, they may be a follow-up (i.e. ineligible) . However, because DO cannot be certain that it is a follow-up smear, then DO must enter all positive smears as .new diagnostic cases.
23. In the event that a patient details was previously entered and the web application indicated that the entry already exist, Data Officer shall enter patient details.

PART 4 GENE XPERT TEST

2. Genexpert is never used for follow-up, so all Xpert test should be considered as diagnostic and all positives recorded.
3. Data officer will extract similar information from gene Xpert register on TB with positive gene Xpert result.
4. In the event that a patient's details appear on both the sputum and gene Xpert register and, the data officer should enter it twice.

APPENDICES: APPENDIX A:

Line listing of Smear Positive TB Cases diagnosed in Lagos Private Laboratories in 2015

Facility name……………………………………………Facility number: ____________ LGA………………………

Date of receipt: ________Name of Data Officer: __________________

Nos	First name	Surname	Age (in years)	Gender M=1 F=2 n/a=77	Address	Type of test (GeneXpert or smear microscopy)	Date of test in 2015	Referring Health Facility

Lagos Inventory Study			
TITLE: Standard Operating Procedures for Facility Entry and Informed Consent			
#005	rev. 00	Effective Date: 22/05/2017	Supersedes rev. NA
Page 1 of 5			
Submitted by: _____ (Signature & Date) Name: Adejumo Olusola Title: Principal Investigator	Reviewed by: _____ (Signature & Date) Name: Title:	Approved by: _____ (Signature & Date) Name: Ellen M.H. Mitchell Title:	

PURPOSE (or OBJECTIVE)

The purpose of this standard operating procedure (SOP) is to describe the responsibilities of the study team as regards to facility entry and The SOP details the procedures to be followed to ensure informed consent and how to manage refusals.

SCOPE

This SOP applies to all study team members participating in research activities for the Lagos Inventory study.

RESPONSIBILITIES

Data Officers are responsible for following this SOP and ensuring the respectful, ethical, and responsive interactions with stakeholders at all levels of the health facility. Oversight of this Standard Operating Procedure (SOP) ensuring that informed consent and facility entry steps are followed at all times: Principal Investigator (PI). Maintaining confidentiality and adhering to this SOP: Data manager and all data officers

Definitions of terms

Confidentiality	Prevention of disclosure, to other than authorized individuals, of a sponsor's proprietary information or of a subject's identity and medical information.
Informed Consent	The process by which a subject voluntarily confirms in writing his or her willingness to participate in a particular study, after having been informed of all aspects of the study that are relevant to the subject's decision to participate.
Investigator	A person who participates in the conduct of the clinical study at a study site. If a team of individuals at a site conducts a study, the Investigator who is responsible leader of the team may be called the Principal Investigator (PI).
Protocol	A document that describes the objective(s), background and rationale for the study design, methodology, statistical considerations and organization of a study.
Record	Any item, collection, or grouping of information that includes protected health information and is maintained, collected, used, or disseminated by healthcare professionals.
Refusal of consent	Voluntary rejection of participation in research after being informed of all aspects of the study that are relevant to the subject's decision to participate
Withdrawal of consent	Rejection to participate in research after giving initial consent
Subject (Participant)	An individual who participates in a research study, either as a recipient of the investigational product or as a control.
FAQ	Frequently asked questions

MATERIALS REQUIRED

1. a letter of introduction from the Lagos State Ministry of Health.
2. Copies of the ethical approval letters from the LSTBLCP and LASUTH
3. data collector identification badge?
4. informed consent forms

NB: The letters of introduction to the sites will be personalized (i.e. with the name and address of the site)and that they be on quality paper and a real signature.. Each letter will be in an envelope also bearing the name and address.

PROCEDURES

The following PARTs will be taken by data officers during verification of TB log books at DOTS facilities.

PART 1: COMMUNITY ENTRY

1. Data officer will ask for the person in charge of the facilities visited and introduce themselves.
2. Data officers will ask for a representative of the health facility if the person in charge is not available.
3. Data officers shall inform the representative or person in charge of the purpose of their visit and hand over the letter of introduction and ethical approval from the LSTBLCP and LASUTH respectively.

4. Data officers must conduct themselves in a way that data collection does not interfere with the activities of the health facility.
5. In the event that the person in charge or representatives of the hospital is busy, data officers shall book an appointment for revisit and inform the data manager of such.
6. To book an appointment for revisit, data officers will ask the person in charge or their representative the convenient date and time for revisit.
7. Data officer will ask for the phone number of the person in charge or their representative for the purpose of sending a reminder before that schedule visit.
8. If the person in charge or representative declined giving his/her phone number, data officers must respect their decision and return to the health facility at the scheduled time.
9. Data officer shall consider that the person in charge of the facility or their representative are unwilling to participate in the study after three visits to the facility.
10. If the person in charge or their representative withdrew consent while data collection is on, the data officer will endeavor to find the reason for the withdrawal and communicate this to the data manager and the PI
11. The PI will endeavor to address the concern of the person in charge or their representative so that data collection could be completed.
12. If the situation could not be remedied, the case shall be taken as withdrawal and shall be noted by the data manager.
13. Data officers should answer questions about the research raised by the person in charge of health facilities visited or their representatives as much as possible. See FAQ below.
14. Data officers should call the Data Manager or the PI when questions they cannot answer are raised on the field
15. Data officers must endeavor not to provide wrong information about the research to the person in charge or representatives of health facilities.

PART 2: INFORMED CONSENT

Steps for obtaining informed consent

1. Data officers shall introduce themselves to the person in charge or their representative and explain the purpose of their visit.
2. Data officer will explain to the person in charge or their representative the purpose of the research.
3. Data officer shall explain the type of data that they want to collect and why they need the data.
4. Data officer will explain to the person in charge or their representative that data collected shall be treated with confidentiality and will not be used for any other purpose than what has been explained to them.
5. Data officer shall explain any potential harm and benefit of the research to the person in charge or their patients.
6. Data officer will explain that participation is voluntary and refusal to participate in the research will not attract any form of sanction from the government.
7. If the person in charge or their representative accept to participate in the study, the data officer must give them the informed consent form to sign.
8. If the person in charge or their representative decides to think over what was discussed, the data officer will leave the informed consent form with the person in charge to go through and shall book an appointment on when to come back (Data officer will collect phone number of the person in charge)
9. In situations when the person in charge or their representatives refuse consent, data officer will persuade them to think carefully about it again ask for their phone number so that data officer can call at an agreed time if the person in charge or their representative has agreed to participate.
10. If the person in charge or their representative still refused consent after one week of visit, the data officer shall consider them to refused consent.
11. All cases of refusal of consent and cases where the person in charge want to think over the research shall be reported by the data officer to the data manager.

<u>Frequently asked questions (FAQ)</u>

1. What is an inventory study
   Answer: Inventory study is conducted to estimate under reporting of TB in a defined geographical area

2. Why do you want to estimate TB under-reporting?
   Answer: To know the accurate picture of the actual TB case detection in the state for planning

3. What do you want to do with the data?
   Answer: We want to know if your data was captured by the Lagos State TB and Leprosy control Program (LSTBLCP) or the Disease Surveillance Notification (DSN) system

4. Are you working with the National or State TB programme?
   Answer: This study was designed in conjunction with the Lagos State TB Program. The National TB program is also aware of this study.

5. What is our own benefit in this research?
   Answer: You are partnering with the state to solve a problem. In addition, you stand a chance of collaborating with the state TB program which may increase your patronage.

6. How can we join the TB program?
   Answer: We will give you the phone number of the contact person for the LSTBLCP

7. Hope you are not looking at the way we manage our patient
   Answer: No, we are not looking at quality of care – only reporting.

8. Question: What is the meaning of KNCV

   Answer: The Royal Dutch Tuberculosis Association. It is one hundred and ten years old. It is the research and policy arm of the Dutch national TB program working in 22 countries to prevent, diagnose, treat TB and restore patients to wellness.
