# Supplementary material for: Hybrid Approach to Estimation of Underreporting of Tuberculosis Case Notification in High-Burden Settings With Weak Surveillance Infrastructure: Design and Implementation of an Inventory Study
Source: JMIR Public Health Surveill. 2021 Mar 15;7(3):e22352. doi: 10.2196/22352 (PMC8088841; doi:10.2196/22352)
Supplement: Multimedia Appendix 4 [file publichealth_v7i3e22352_app4.docx]

**Supplementary File 4: comparison of the structure and case counts of the TB and Infectious Disease Surveillance Systems in Nigeria in 2015**

It is potentially enlightening to compare HF notification of TB cases to STBLCO with TB notification to the parallel state-wide monthly disease reporting of the IDSR because this system captures TB treatment regardless of “engagement” status. The state TB program (STBLCO) is the authoritative department within Lagos State Ministry of Health on matters of TB surveillance.

#### Table 1: Comparison of Lagos State TB Notification Systems

|  | STBLCP | IDSR |
| --- | --- | --- |
| Collected by | TBLS | IDSR |
| Number of facilities eligible to notify TB cases | 315 | 2,253 |
| Units counted | Cases | Services rendered |
| Periodicity | Quarterly | Monthly |
| Age aggregation | 8 brackets (2 pediatric) | 7 brackets (5 pediatric) |
| Sex disaggregated | yes | yes |
| Case-based | yes | no |
| Unique IDs | no | no |

All facilities are expected to submit a monthly report. Routine monthly notification form: IDSR 003 should be submitted by all facilitates in the state. The STBLCO system is “active” in that notifications are actively solicited from sites whereas IDSR is “passive”, in the sense of routine reports are pushed to the IDSR.

The structure of the IDSR data are monthly sex and age disaggregated facility case counts for inpatient TB admissions and outpatient TB diagnoses. The system does not include TB type, means of diagnosis, and treatment information or TB treatment outcomes. Cases are reported as inpatient or outpatient and may therefore contain duplicates. The potential for duplicates, the lack of required TB cohort surveillance variables, and highly variable participation by facilities has limited the use of these data by the STBLCO.

#### Table 2: Comparison of Facility Participation in Parallel Surveillance Systems

|  | **STBLCO**  **2015** | **IDSR**  **2015** |
| --- | --- | --- |
| Collected by | Actively transcribed by TBLS | Passively received by IDSR officer |
| Number of HF eligible to notify TB cases through this system | 315 | 2,253 |
| Number of HF classified as active in 2015 (% of total) | 315 (100%) | 426 (18.9%) |
| Number of HF that reported ≥ 1 TB cases in 2015 (% of facilities participating) | 258 (83.9%) | n/a |
| Total TB cases notified | 8,770 | 6,301 |

Participation in IDSR reporting varied by LGA, with over 50% of facilities contributing TB data in Ibeju Lekki, Epe, Ikeja, Shomolu, and Ikorodu. One LGA (Ifako/Ijaye) contributed no notifications for any disease during 2015. Although the number of HF (n=426) expected to contribute disease notifications to the IDSR is greater than the number providing notifications to the STBLCO (n=315), the proportion of HF notifying TB in 2015 was substantially lower (18.9% vs 83.2%) (See Figure 7).

Figure 1: Compliance with IDSR reporting by LGA

High variability in compliance behavior and facility density among LGA make the TB notifications for 2015 challenging to interpret. The IDSR notified 5,608 TB out-patients and 6,301 TB in-patients from 426 participating facilities in 2015. Five LGA (Ibeju Lekki, Lagos Mainland, Ojo, and Surulere) did not contribute any TB notifications to the IDSR system during 2015. One LGA, Shomolu, reported 1,359 TB outpatients treated, while the LGTBLS reported 262 TB cases to the STBLCP. The proportion of facilities contributions to the IDSR varied widely.
